# Design and Implementation of High-Precision Vision Measurement Automatic Inspection Platform for Intelligent Manufacturing

**DOI:** 10.3390/s26216984

**Published:** 2026-11-05

**Authors:** Lulu Huang, Mingyang Yang, Zepeng Ni, Dongmei Gong, Kuai Zhou, Peng Zhao, Feng Xu

**Affiliations:** 1 School of Mechanical and Vehicle Engineering, West Anhui University, Lu’an 237012, China; huanglulu@wxc.edu.cn (L.H.); 2025210015@mail.wxc.edu.cn (M.Y.); 2026210015@mail.wxc.edu.cn (Z.N.); 04000031@wxc.edu.cn (D.G.); 2 School of Engineering, Anhui Agricultural University, Hefei 230036, China; 3 School of Aeronautical Engineering, Nanjing University of Industry Technology, Nanjing 210023, China; 2024101500@niit.edu.cn; 4 College of Mechanical and Electrical Engineering, Nanjing University of Aeronautics and Astronautics, Nanjing 210016, China; zhaopeng@nuaa.edu.cn

**Keywords:** smart manufacturing, visual measurement, automated inspection, registration analysis

## Abstract

In response to the demand for efficient automated inspection of parts in the field of intelligent manufacturing, this paper proposes a design method for a high-precision visual measurement automated inspection platform oriented toward intelligent manufacturing scenarios. First, specifications for the layout of fixture reference points were established. An automatically rotating fixture was designed, and a robotic system was integrated with precision measurement equipment to build an integrated inspection and analysis platform. Robot collision simulation analysis and measurement registration analysis were completed, and key parameters in the inspection process were analyzed in light of the diverse inspection requirements of smart manufacturing. An automated measurement and analysis system that integrates automated testing, data processing, and analysis has been developed. Finally, validation tests were conducted. The results demonstrate that the automated inspection platform developed herein can efficiently implement automated product inspections with favorable measurement repeatability and high inspection efficiency, improving inspection performance for smart-manufacturing applications. The proposed method has good versatility and adaptability and can offer technical support for automated inspection in smart manufacturing applications.

## 1. Introduction

In recent years, high-precision 3D scanning and measurement technology has been widely applied in many fields, including aerospace, automotive, healthcare, and cultural heritage restoration, thanks to its unique advantages [1,2,3,4,5]. In actual engineering applications, numerous issues still exist; for example, factors such as the inherent characteristics, geometric dimensions, and surface coatings of the object under test can all affect measurement accuracy and quality to varying degrees. To address measurement challenges in various scenarios, researchers have conducted extensive studies in areas such as measurement error optimization, feature recognition, detection efficiency, point cloud data processing, and scan path and robotic arm trajectory planning, achieving significant results. Regarding factors affecting inspection quality, such as surface coatings and microscale features on the test workpiece, Duenser and Han et al. [6,7] addressed these challenges successfully using a weighting strategy and the discrete wavelet transform algorithm. Chen and Wu et al. [8,9] developed an integrated framework that combines autonomous navigation with point cloud analysis to address certain surface flatness measurement problems, thereby enabling point cloud segmentation and flatness analysis. Point cloud data is critical to ensuring the accuracy of 3D scanning measurements. To address the issue of excessive noise in measurement data caused by end-effector vibration, Li et al. [10] established a mapping relationship between vibration and noise by combining variational modal decomposition with vibration response, effectively suppressing point cloud noise and ensuring the continuity of the surface profile. In terms of parameter calibration and feature extraction, to address issues such as the lack of image features in line-scan structured light sensors and the cumbersome calibration process, Zhong et al. [11] proposed a simple and stable calibration method that combines a two-step iterative algorithm with a fast simulated annealing algorithm under single-plane constraints, thereby improving the stability and convenience of the calibration process. To address the challenge of feature extraction caused by sparse point clouds, Yu et al. [12] developed a single-stage 3D object detection framework. They designed a multi-scale voxel alternating fusion module to generate a more adaptable 3D object feature representation. To address the issue of incomplete point clouds for the product under test caused by equipment obstruction, Hong et al. [13] developed an attention-enhanced point cloud patching network that integrates a curvature-weighted sampling feature extraction module with a spatial attention mechanism. This approach enhances the network’s sensitivity to geometric structural differences, enabling precise reconstruction of the point cloud. To address the issue of poor local feature distinguishability in point cloud segmentation methods, Cheng et al. [14] proposed a lightweight point cloud classification and segmentation network with adaptive feature extraction, which achieves adaptive feature enhancement through geometric-topological learning. In addition, Zhao et al. [15] developed a detection framework based on monocular depth estimation and a dual-attention point cloud network, incorporating both channel and spatial attention modules to enhance the representation of key features and thereby improve point cloud detection performance.

Scan path planning is a key method for improving the accuracy, efficiency, and scene adaptability of 3D scanning. To address the kinematic constraints associated with inspecting large free-form surfaces, Pi et al. [16] proposed a scan path planning method specifically designed for robotic optical measurement systems, which achieves intelligent path optimization through a curvature-adaptive generation strategy. Considering that the pose of the product under test and the scanning distance can affect measurement accuracy, Malhan et al. [17] proposed a robot motion planning method that incorporates camera performance constraints. By capturing a point cloud of the part using a camera and optimizing the sparse point cloud distribution through offline planning, this method ensures the uniformity of the part’s overall point cloud. To reduce dependence on geometric models, Kaiser et al. [18] introduced a simulation environment based on reinforcement learning to model detection behavior, enabling the efficient solution of planning problems under operating conditions without a predefined geometric model. To meet the demand for high-precision, high-efficiency batch scanning of components, researchers have developed automated scanning systems based on path planning, which offer superior overall performance in areas such as parameter calibration, path calculation, and multi-view point cloud registration [19,20]. Existing research on path planning has largely focused on vision-based robots with fixed bases, resulting in limited versatility. In response, Kong et al. [21] developed a coverage-based path planning model that combines allocation and sequence optimization, thereby improving measurement efficiency. Chen et al. [22] proposed an adaptive path planning method based on surface geometric features, which generates sampling points based on surface characteristics and solves for the optimal scanning path using dynamic programming, effectively addressing the issue of low measurement efficiency for free-form surfaces.

Robotic arm collaborative operations have been widely adopted in fields such as aerospace, agriculture, and industry, and robotic arm path planning is critical to the quality and efficiency of these collaborative operations [23,24,25,26]. Researchers have optimized robotic arm trajectories under multiple constraints, including motion smoothness, optimal trajectories, and machining accuracy [27]. To address the need for collision-free path planning in confined spaces and environments with dense obstacles, Elhadidy et al. [28] developed a novel hybrid meta-heuristic algorithm that combines the global search capabilities of the Whale Optimization Algorithm with the local exploration advantages of the Genetic Algorithm, thereby reducing path length while ensuring smooth motion. To address the challenge of optimal path planning for high-degree-of-freedom robotic arms, algorithms such as the improved bidirectional fast-expanding random tree algorithm, genetic algorithms, and the whale optimization algorithm were employed. By optimizing motion paths in accordance with the robotic arm’s constraints, the planned paths became more reasonable and efficient [29,30]. To address the challenges of machining large-sized, low-stiffness, and easily deformable components, researchers have proposed an optimization objective function that focuses on path smoothness to eliminate the effects of local discontinuities while also establishing a robot posture optimization objective function to enhance the stability of robot operation [31,32]. To address the challenges of machining complex surfaces, Xiao et al. [33] developed a numerical contact mechanics model for complex workpieces based on point sampling, which accurately predicts the contact pressure distribution on the workpiece’s point cloud surface and effectively improves the machining accuracy of complex workpieces.

In response to the demand for high-precision automated inspection in the field of smart manufacturing, this paper proposes a design method for a high-precision 3D vision-based automated inspection platform tailored to smart manufacturing scenarios. An automatic rotating fixture was designed, integrating industrial robots and precision measurement equipment to establish an integrated inspection and analysis platform. Additionally, a software system combining automated inspection, data processing, and result analysis was developed, effectively improving the measurement accuracy and inspection efficiency of smart manufacturing products. The content of each section in this paper is organized as follows: Section 2 addresses the requirements for automated inspection by completing the structural design of an automatic rotating fixture and the setup of a robotic system, followed by a registration analysis. Section 3 begins by establishing specifications for the layout of measurement points on tooling and completing a robot collision simulation analysis for the automated inspection platform. Taking into account the diverse inspection requirements of smart manufacturing, it analyzes the core parameters involved in the inspection process. Finally, it completes the development of automated inspection analysis software. Section 4 describes the construction of a complete automated 3D vision measurement and analysis system and verifies the platform’s inspection performance through experiments. The experimental results demonstrate that the high-precision automated 3D vision inspection platform design proposed in this paper can efficiently perform automated measurement and analysis tasks, possesses good versatility and adaptability, and can be widely applied in various smart manufacturing inspection scenarios. Finally, Section 5 summarizes the research conclusions of this paper.

## 2. Overall Design of an Automated Inspection Platform

### 2.1. Requirements for Automated Measurement

At the platform integration level, several cyber–physical frameworks have been developed to fuse sensor data and digital models for aerospace manufacturing. One representative example is the XR- and DSL-enabled CPS framework reported in [34]. This work adopts a domain-specific language to formalise and standardise assembly instructions, combining object recognition, target tracking and real-time sensor inputs to deliver immersive XR guidance for operators throughout aerospace AIT operations. Its performance was validated on CubeSat assembly trials, demonstrating reduced human error alongside gains in task efficiency and usability. Although our work similarly pursues close integration of robotic hardware, optical sensors and metrology software, the intended function and underlying integration logic diverge markedly. Our platform is built for autonomous robotic 3D geometric inspection, rather than XR-supported manual assembly. Accordingly, our integration priorities centre on collision-free viewpoint planning, unified multi-sensor coordinate systems and repeatable quantitative deviation analysis, instead of interpreting assembly scripts and generating augmented visual overlays for on-site technicians.

**Figure 1:**
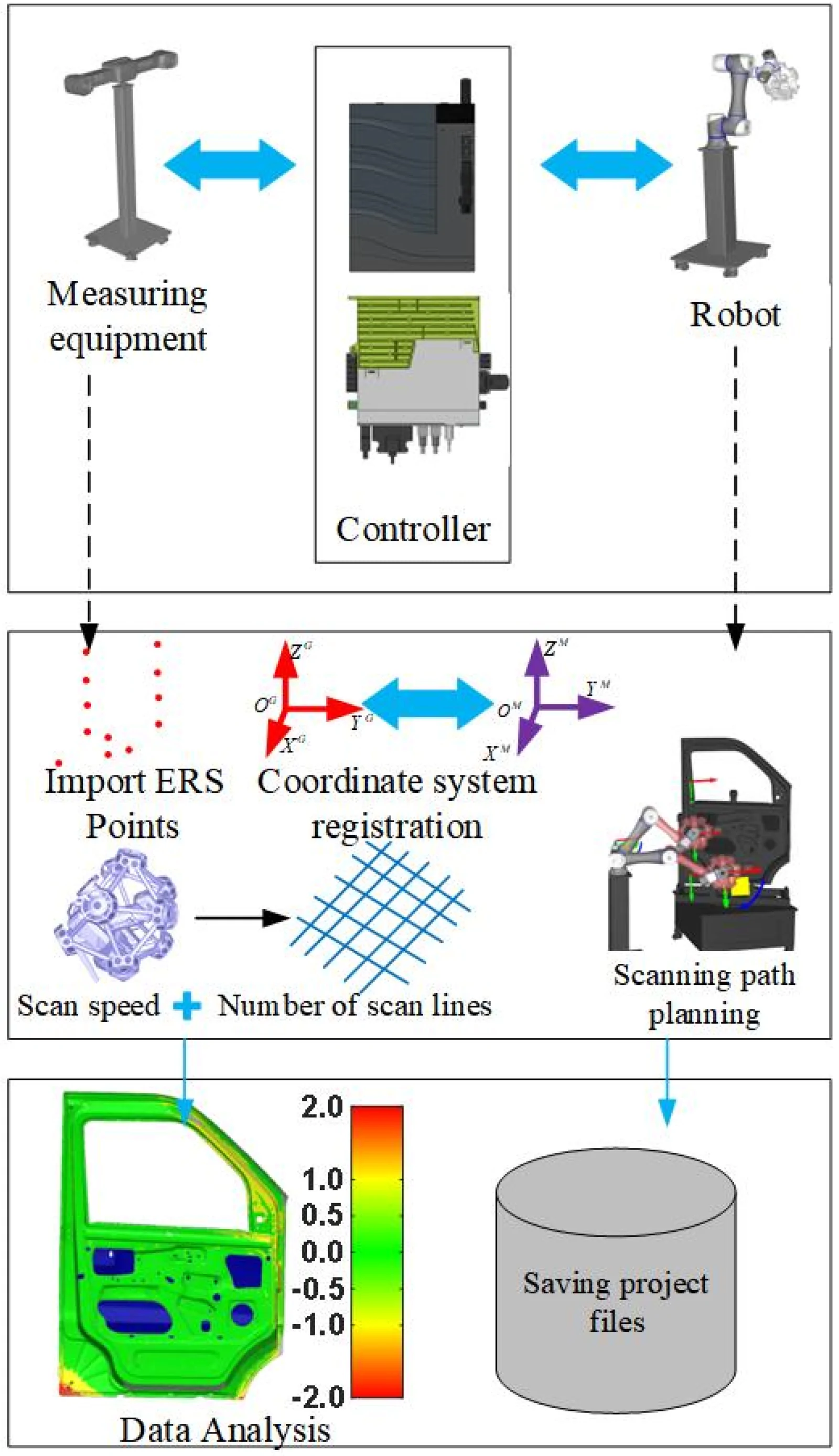
Overall design of an automated inspection platform.

To meet the demands of smart manufacturing and improve product inspection efficiency, this paper designs a dedicated rotary fixture independently. On this basis, the proposed system integrates commercially available robotic systems and 3D vision measurement equipment to build a complete inspection hardware platform. Furthermore, this paper proposes a 3D vision-based automated inspection method tailored for smart manufacturing and finally develops a supporting 3D vision-based automated inspection and analysis software system. The proposed workflow supports automated product inspection, reduces human-dependent operations, and improves inspection efficiency. The automated inspection platform consists primarily of the self-designed rotary fixture base, integrated commercial robotic system, and 3D vision measurement equipment; the overall design of the automated inspection platform is shown in Figure 1.

### 2.2. Workpiece Fixture Design

**Figure 2:**
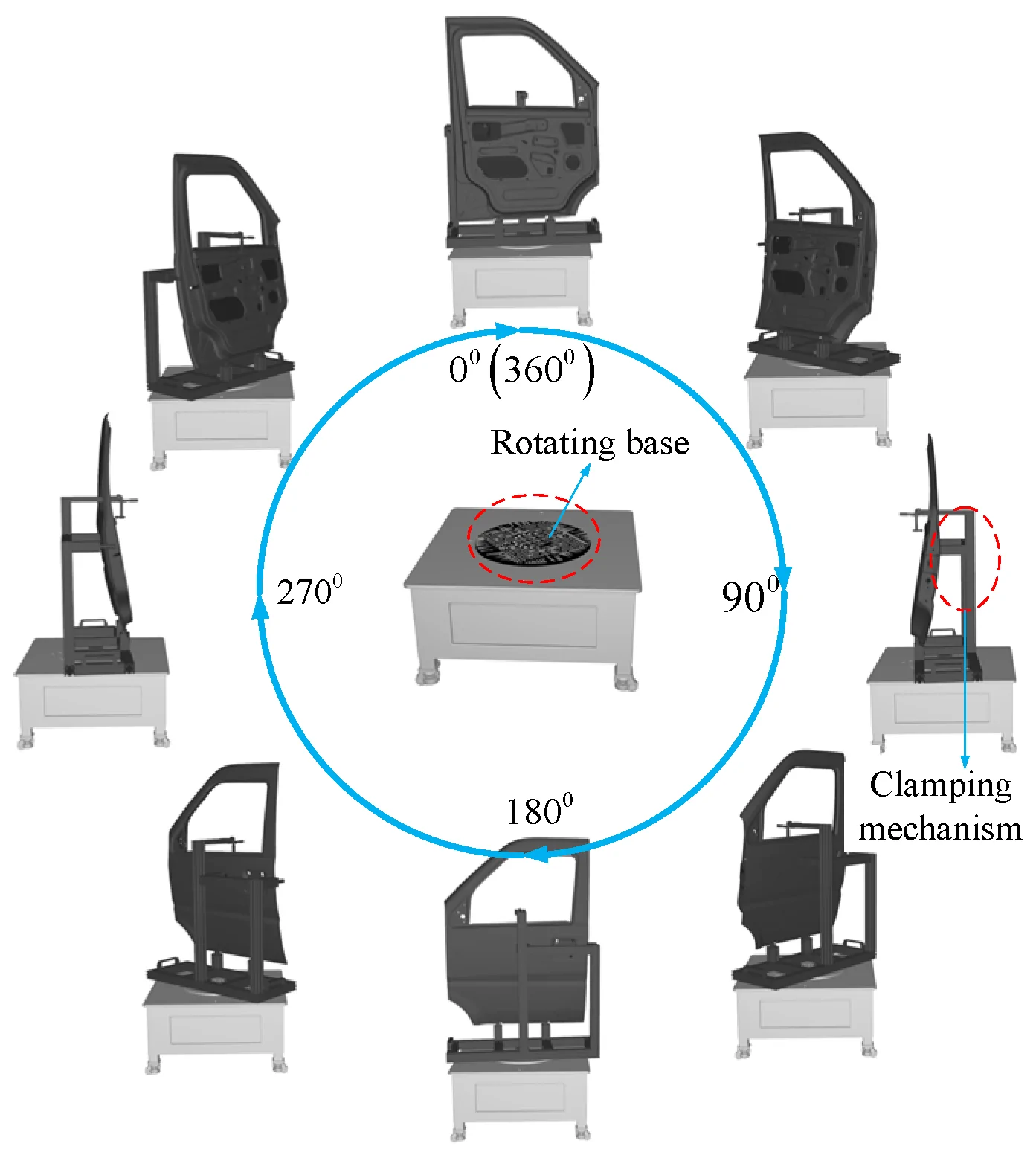
Fixture base structure.

To realize fully automated multi-directional measurement of parts, it is necessary to consider not only multi-angle scanning but also autonomous rotation of workpieces. To address this requirement, this paper proposes a rotatable fixture base, as illustrated in Figure 2. As the core supporting component of the 3D vision automated inspection platform, this fixture serves a vital function in the automated measurement workflow. The rotary fixture base delivers a stable, precise foundation for clamping and positioning test workpieces. Based on the category and structural features of the workpiece, customized clamping and positioning mechanisms can be designed for the rotary base to securely mount products with diverse specifications and geometries. This avoids workpiece displacement or vibration during measurement and guarantees reliable positioning for subsequent high-precision automated measurements.

This paper describes a rotating fixture base capable of rotating 360°, which expands the measurement field-of-view and maximizes the accessible measurable outer-surface area of the component. Complete measurement coverage cannot be achieved for features occluded by clamps or deep enclosed internal cavities, because optical laser scanning strictly relies on line-of-sight access; such occluded geometries cannot be directly sampled. During 3D visual scanning, the rotary fixture orients clamped workpieces to supply multi-angle scanning perspectives for the 3D measurement system. This mitigates the limitations of static fixed fixtures and improves coverage over exposed outer-surface features. Nevertheless, surfaces blocked by clamping hardware and deep enclosed cavities remain optically inaccessible. Measurement data can only cover optically exposed key outer-surface features rather than all geometric features of the component.

### 2.3. Robot System Setup

**Figure 3:**
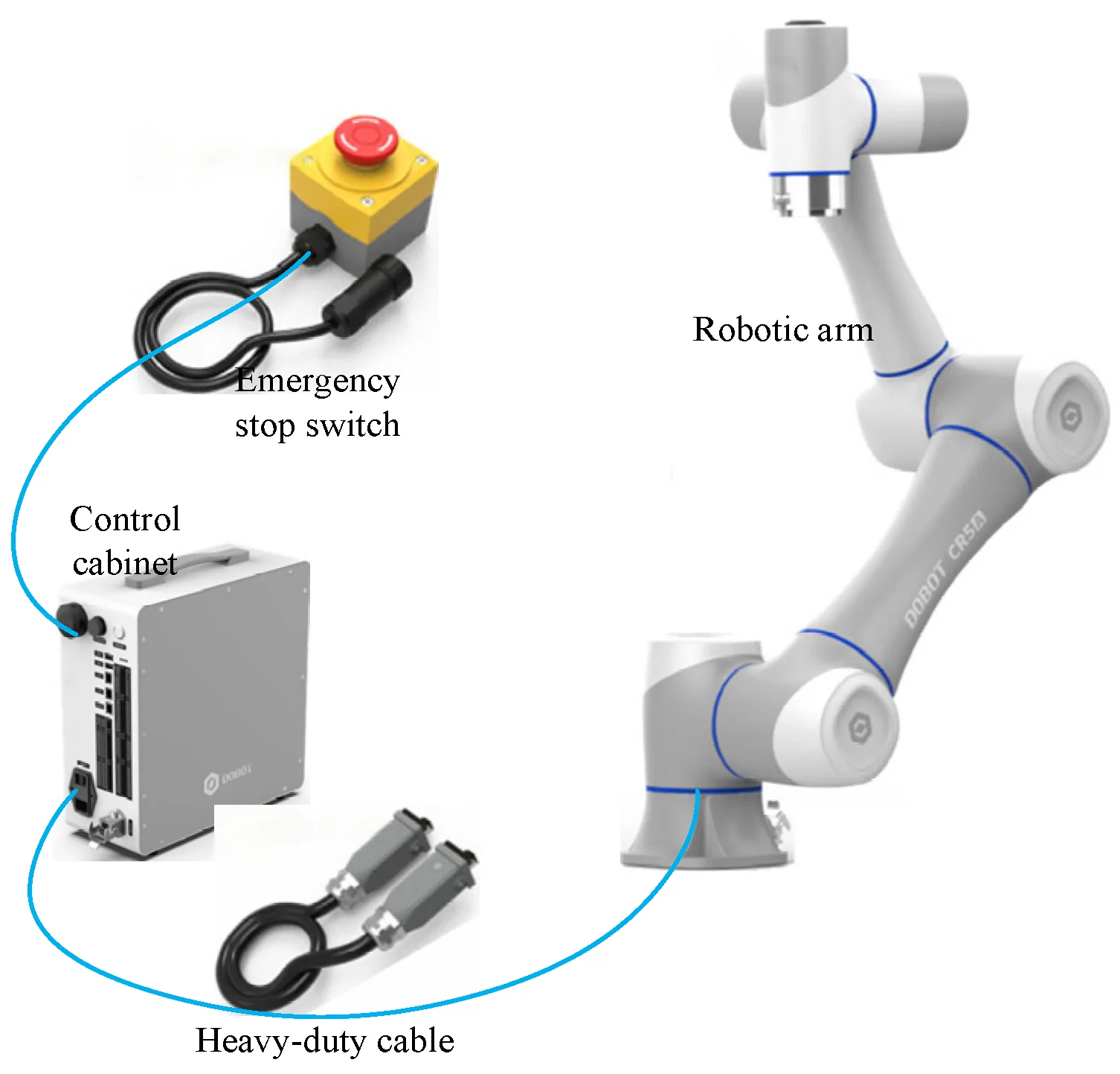
Dobot CR5A 6-axis robot system.

To expand the measurement range of 3D vision measurement equipment and enhance its measurement flexibility and applicability, this paper uses a 6-axis robotic arm, as shown in Figure 3, as the mounting and motion execution mechanism for the 3D vision measurement equipment. The 6-axis robotic arm features multi-degree-of-freedom motion, enabling the 3D vision measurement system to rotate flexibly along different spatial coordinate axes, thereby overcoming the spatial limitations imposed by fixed installations. When combined with the rotating fixture designed in this paper, it can easily cover all surfaces of the product under test, effectively resolving issues such as insufficient measurement range associated with a single, fixed measurement perspective.

Following the pre-defined measurement path program, the robot adjusts the spatial pose of the scanning device to realize automated workpiece measurement and removes the requirement for manual pose adjustment. The robotic arm’s positioning performance stabilizes scanner posture during motion and supports consistent measurement data acquisition. Its configurable motion trajectories also accommodate workpieces of varying dimensions and geometries, improving platform versatility for automated-inspection tasks. Specific parameters are shown in Table 1.

Robot forward kinematics derives the position and orientation of the end-effector based on the given joint angles of each robot joint. Using the D-H method, the link transformation matrix for the adjacent coordinate system of a robotic arm can be established as follows [35]:

(1) Ti  i−1=Rot(zi−1,θi)Trans(0,0,di)Trans(ai,0,0)Rot(xi,αi)=cosθi−cosαisinθisinαisinθiaicosθisinθicosαicosθi−sinαicosθiaisinθi0sinαicosαidi0001

**Figure 4:**
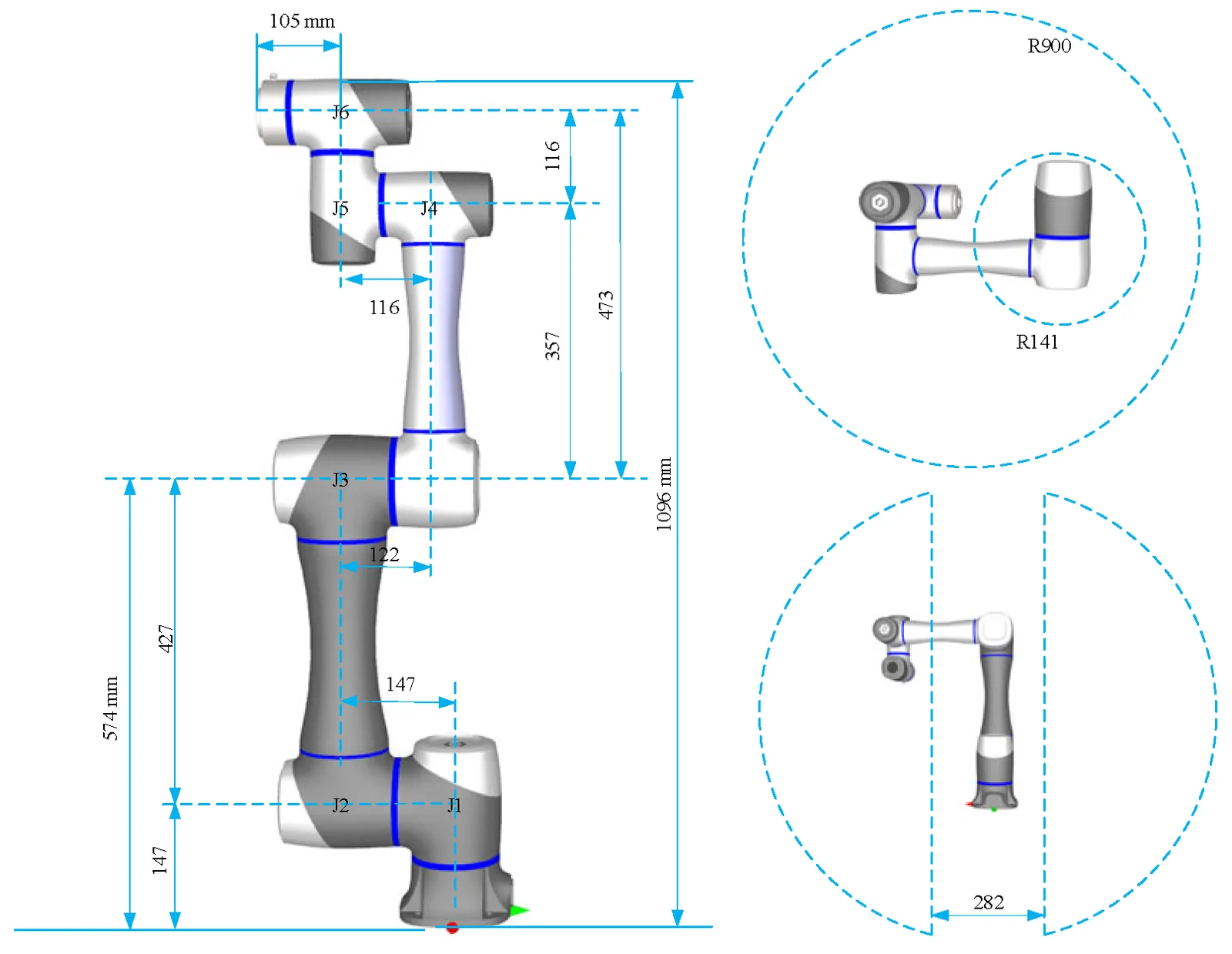
Robot parameter information.

Here, *Rot*(*z_i_*_−1_,*θ_i_*) represents a rotation of angle *θ_i_* about the *z_i_*_−1_-axis, with counterclockwise being the positive direction and clockwise being the negative direction. *Trans*(0,0,*d_i_*) represents a translation of distance *d_i_* in the positive direction along the *z_i_*-axis. *Trans*(*a_i_*,0,0) represents a translation of distance *a_i_* in the positive direction along the *x_i_*-axis. *Rot*(*x_i_*,*α_i_*) represents a rotation of angle *α_i_* about the *x_i_*-axis. Substituting the dimensions of each joint of the robotic arm (Figure 4) into Equation (1) yields the end-effector pose matrix as follows:

(2) T60=10T21T32T43T54T65T=nxoxaxpxnyoyaypynzozazpz0001

where the 3D matrix $\left[\begin{array}{ccc} n & o & a \end{array}\right]$ and vector *p* represent, respectively, the pose and position of the end-effector relative to the base coordinate system in the three coordinate directions. Robot inverse kinematics involves determining the rotation angles of each joint of the robotic arm given the known pose of the end-effector relative to the base coordinate system. Robot inverse kinematics can be solved using the projection method and the analytical method [36].

### 2.4. High-Precision Visual Measurement and Registration Analysis

**Figure 5:**
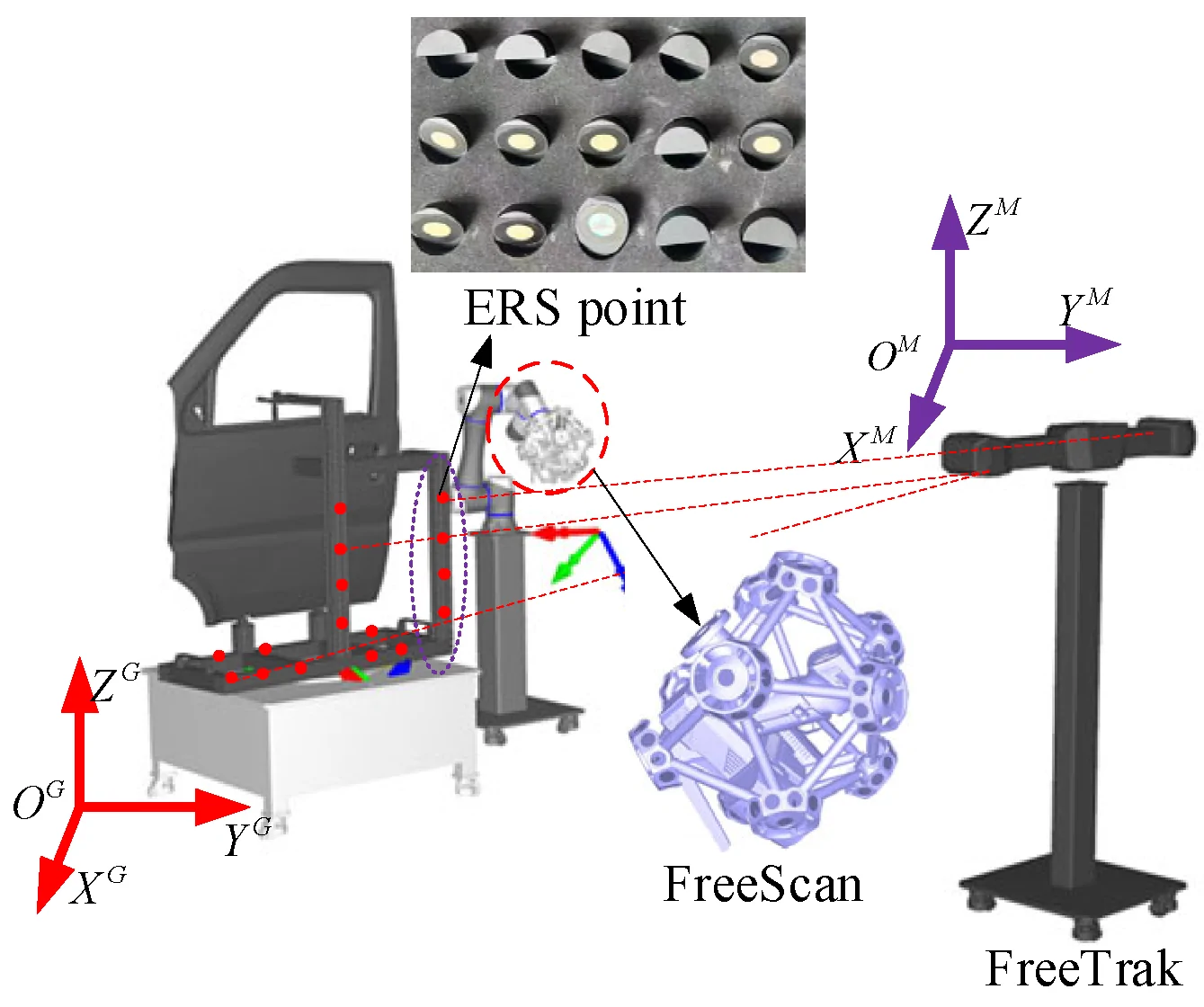
3D visual measurement system.

Vision measurement systems serve as core components for automated high-precision 3D inspection and play a vital role in automated measurement workflows. Leveraging multi-view imaging and 3D reconstruction techniques, such systems can capture the spatial coordinates, contour features, and surface information of workpieces without physical contact or damage. This capability fulfills the high-precision and high-efficiency inspection demands of smart manufacturing. The automated inspection platform described in this paper utilizes the high-precision measurement equipment shown in Figure 5. This equipment employs a robotic arm end-effector to secure a 3D laser scanner for automated scanning operations. At the same time, an optical tracker tracks the 3D laser scanner and reference points mounted on the measurement fixture to enable real-time tracking of measurement data. The fixture structure itself has its own coordinate system, denoted as the global coordinate system {*O^G^* − *X^G^Y^G^Z^G^*}, while the position of the 3D scanning device is the measurement coordinate system, denoted as {*O^M^* − *X^M^Y^M^Z^M^*}. The reference points established on the fixture structure are measured and denoted as *P^M^* = {*p_i_* = (*x_i_*,*y_i_*,*z_i_*),*i* = *m*}.

Both the measurement equipment and the product under test have independent coordinate systems, and no relationship has been established between these coordinate systems. To establish a connection between the data in different coordinate systems, calibration is performed using the measurement data and the initial data according to Equations (3) and (4), and the coordinate system rotation parameters *R* and translation parameters *t* are obtained when the residuals are minimized [37].

(3) $$P^{G}=RP^{M}+t$$

(4) $$E^{2}=\frac{1}{n}\sum_{i=1}^{n}{\left\|RP^{M}+t-P^{G}\right\|}^{2}$$

(5) $$R=\left[\begin{array}{ccc} \cos\alpha_{z} & -\sin\alpha_{z} & 0\\ \sin\alpha_{z} & \cos\alpha_{z} & 0\\ 0 & 0 & 1 \end{array}\right]\left[\begin{array}{ccc} \cos\alpha_{y} & 0 & \sin\alpha_{y}\\ 0 & 1 & 0\\ -\sin\alpha_{y} & 0 & \cos\alpha_{y} \end{array}\right]\left[\begin{array}{ccc} 1 & 0 & 0\\ 0 & \cos\alpha_{x} & -\sin\alpha_{x}\\ 0 & \sin\alpha_{x} & \cos\alpha_{x} \end{array}\right]$$

(6) $$t={\left[t_{x},t_{y},t_{z}\right]}^{T}$$

where *P^G^* denotes the initially calibrated ERS point data of the measurement fixture, and *P^M^* represents the measured data. *α_z_* is the axis’s rotation angle around the Z-axis, *α_y_* is the axis’s rotation angle around the Y-axis, and *α_x_* is the axis’s rotation angle around the X-axis.

**Figure 6:**
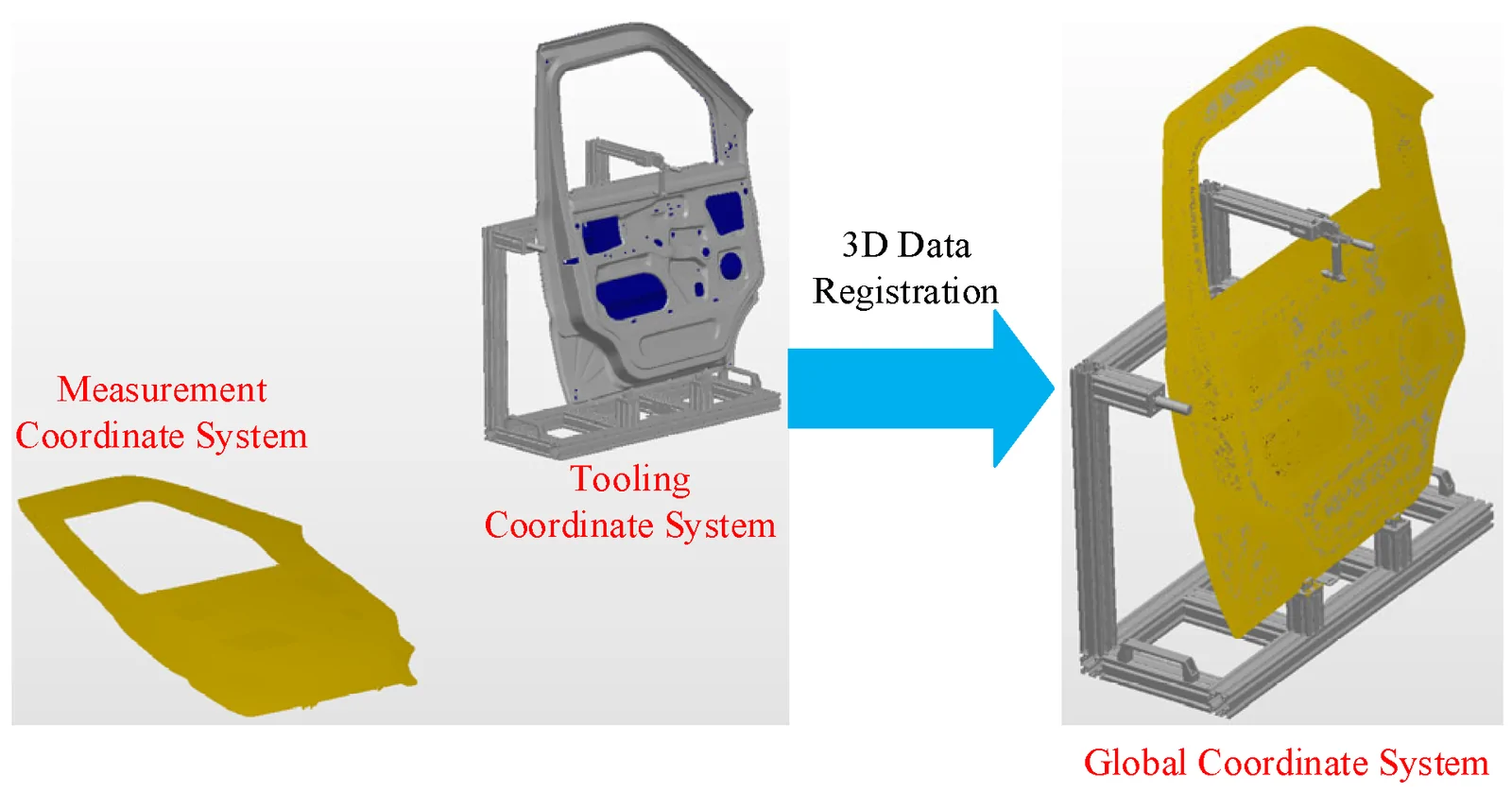
Data registration across different coordinate systems.

As shown in Figure 6, the theoretical model data of the target under test are in the fixture coordinate system, while the measured data are in the instrument’s own measurement coordinate system; the data from these two systems cannot be directly used for data analysis. Therefore, after obtaining the optimal coordinate system parameters, the measured data can be transformed into the global coordinate system using Equation (3), thereby enabling direct registration between the target data and the geometric model, which facilitates subsequent deviation analysis.

The proposed fixture mounts the measurement sensor firmly on the robot end-effector. After attachment, the scanner holds a steady pose to capture surface features of the test workpiece. This arrangement reduces variability inherent to manual scanning and helps produce repeatable measurements. The platform coordinates the vision sensor, robotic manipulator, and rotary fixture base. It executes automated scans and outputs data following pre-planned trajectories and measurement strategies, enabling end-to-end automated workflows from workpiece fixturing to data capture.

## 3. Key Parameters of an Automated Measurement Platform

### 3.1. Layout of Survey Control Points

**Figure 7:**
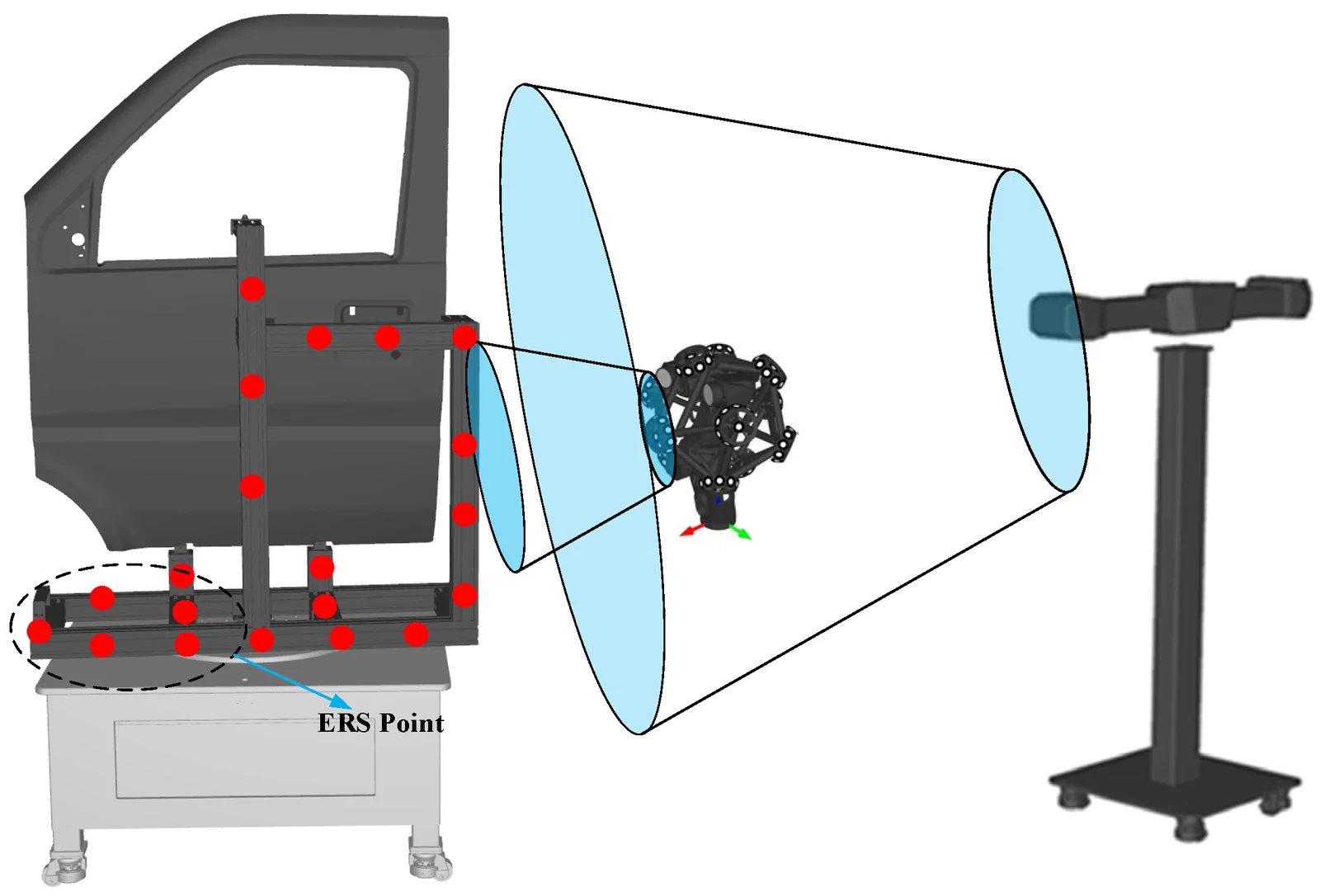
Layout of survey control points.

To ensure that the laser from the measuring equipment covers the product under test, that the tracking device can easily follow the product, and that data continuity is maintained, measurement reference points must be strategically placed. At the same time, it is necessary to ensure that the tracking device can track the reference points even when the rotating base is positioned at any angle during the automatic measurement process. To achieve this, measurement reference points must be strategically placed within the fixture structure. The fixture structure, positioners, and the product under test may obstruct the light source of the measurement equipment, preventing the equipment from directly measuring the reference points. To address this issue, in addition to the reference points laid out on the fixture, a redundant design based on the fixture’s structure is required to additionally place Enhanced Reference System (ERS) points and target points to cover the entire assembly fixture. This ensures a sufficient number of common measurement points, facilitating coordinate system registration. The reference points and target points serve the same function and purpose, which are collectively referred to as ERS points in this paper, as shown in Figure 7.

To ensure measurement accuracy, all ERS points should enclose the entire measurement space. When a measurement point is located within the space enclosed by the established reference points, the measurement error is less than the maximum measurement error of the established reference points. The laid-out reference points must enclose the entire measurement space to facilitate measurement and equipment tracking. The three-dimensional space formed by the measurement reference points should enclose the measurement object, and the geometric center of the reference envelope should be as close as possible to the geometric center of the object being measured. The measurement object designed for enclosure should include features within the fixture that require measurement. Based on the fundamental theory of rigid-body matching, when performing registration measurements, a measurement device only needs to select three non-collinear reference points within the assembly measurement space to participate in the registration analysis. However, to improve the accuracy of registration when moving between measurement stations, three or more reference points are generally selected to complete the registration analysis and achieve coordinate system transformation [38]. To ensure that the tracking device can measure the reference points regardless of the fixture’s rotational position, the layout of reference points in the measurement space should follow the principle of redundancy, and the reference points should be arranged to cover the entire measurement space. Reference points, target measurement features, and measurement equipment should be mounted on relatively stable structures to ensure the stability and consistency of measurement points and their coordinate values throughout the measurement cycle or scheduled inspection cycle. Reference points should be mounted on stable fixture structures, and the stiffness of the reference support structures should be analyzed. The fixture’s own structure must ensure rigidity and stability. Reference points should undergo stability analysis and evaluation to assess their drift status over the scheduled inspection cycle. In principle, the fixture base or independent measurement structure containing the reference point layout should be isolated from personnel and material passageways. Reference points should not be placed on components with poor stability, removable parts, or moving bases. The initial calibration coordinates of all control points laid out in the measurement area must be determined within a unified reference coordinate system. This ensures the consistency of the control point data and provides a unified data foundation for subsequent coordinate system construction and registration analysis.

### 3.2. Robotic Arm Path Planning Simulation

To address the demand for automated product inspection, this paper proposes a robotic arm trajectory planning method for the workpiece that integrates global coverage with local refinement. The global coverage trajectory combines area scanning with bow-shaped scanning strategies. Based on the workpiece structure, the part is partitioned into multiple work zones, within which parallel Z-shaped scanning paths are generated while the overlap between adjacent paths is controlled.

In contrast to the planners cited in the literature, which predominantly rely on fixed global paths or manually predefined high-resolution regions, the proposed method automatically identifies refinement regions according to geometric curvature. Following the preliminary global scan, surface curvature is computed from the acquired point cloud data. When the local curvature exceeds a preset threshold, local refinement is triggered. The scan spacing and the number of scanning lines within a refined zone are determined by the curvature magnitude: a higher curvature yields a smaller scan spacing and a greater number of scan lines, enabling the capture of fine geometrical details. Local refinement terminates once the entire feature region is fully covered or the maximum permitted number of local scan lines is reached.

Most previously reported robotic scanning planners in the cited references adopt either manually predefined regions of interest for high-density scanning or a fixed uniform scan spacing across the entire workpiece surface. In contrast, the proposed two-layer planning framework eliminates manual marking of feature regions. Refinement zones are automatically extracted based on surface curvature calculated from the preliminary global point cloud, which avoids subjective human intervention and reduces pre-scan preparation workload.

Local refinement is triggered when the local surface curvature calculated from the initial global scan point cloud exceeds the preset threshold of 0.02 mm^−1^. The scan spacing and the number of scan lines are determined quantitatively according to the magnitude of local curvature, and all spacing values are constrained within the adjustable range of the FreeScan Trak ProL (0.01–10 mm). In this work, the global Z-shaped scanning adopts a fixed spacing of 2 mm. Within refined regions, scan spacing decreases linearly with rising curvature, with a lower limit of 0.1 mm. The number of scan lines allocated to each refined patch increases correspondingly and is capped at 20 lines for a single local region. Local refinement terminates once either of the two criteria is satisfied: (1) the whole high-curvature region is fully covered by planned scanning trajectories or (2) the predefined upper limit of 20 scan lines is reached, preventing uncontrolled growth of scanning time. These rules are consistent with the feature selection and counting logic summarized in Table 2.

The trade-off among surface coverage, detail resolution and cycle time is realized through this hierarchical sampling strategy. The coarse global scan at 2 mm spacing guarantees complete geometric coverage of the workpiece and establishes the baseline inspection duration. Dense sampling with spacing down to 0.1 mm is only assigned to automatically identified high-curvature features rather than the whole surface. The curvature threshold, spacing bounds and maximum scan-line count serve as adjustable parameters. Increasing the curvature threshold reduces the area requiring refinement and shortens the cycle time at the expense of local geometric resolution. Decreasing the threshold enables capture of finer surface features but prolongs total inspection time.

**Figure 8:**
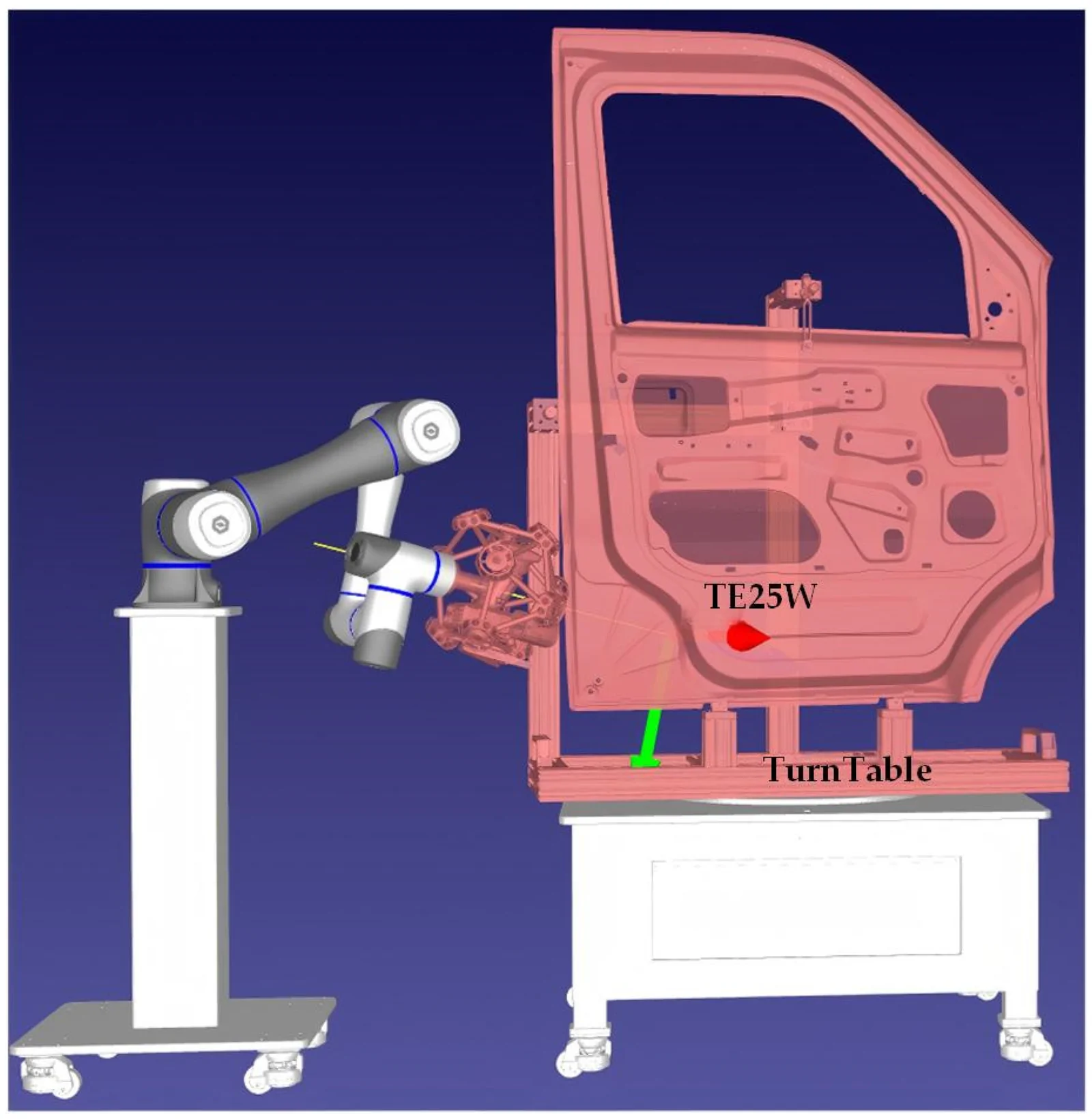
Crash simulation analysis.

A collision simulation environment was established in RoboDK by importing precise geometric models of the robotic arm, end-effector, measurement equipment, workpiece, and fixture. The homogeneous transformation relationships across all coordinate systems were unified, and a high-precision collision mesh was configured. RoboDK’s collision-detection function was then used to analyze trajectory collisions, as illustrated in Figure 8. Through this collision simulation, measurement points were predefined and measurement paths were planned in advance to complete the simulation-based evaluation. This strategy effectively avoids equipment collisions during physical measurement and guarantees measurement continuity.

**Figure 9:**
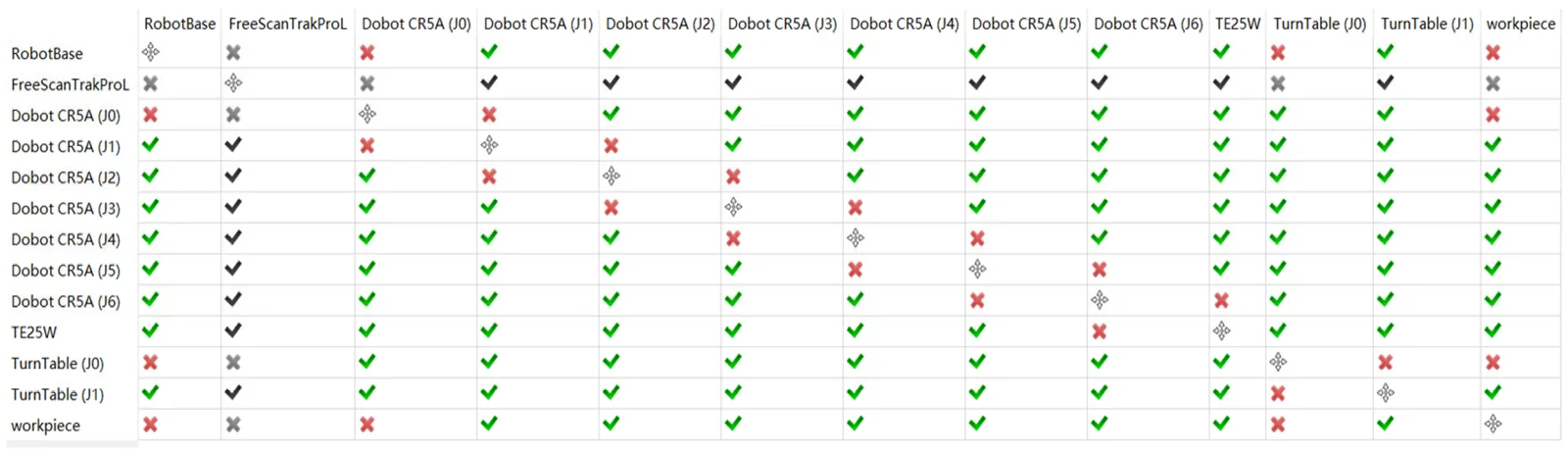
Robot arm joint collision map in the event of a collision.

Figure 9 shows the joint collision map for the robotic arm 3D scanning system. This matrix maps all of the system’s moving linkages and fixed components as its rows and columns, respectively. It allows for the quick and intuitive determination of spatial interference for any given component. It serves as the core basis for collision-free trajectory planning, collaborative operation configuration optimization, and joint limit setting for the robot.

This collision detection covers 12 spatial units within the system, including the RobotBase, FreeScan Trak ProL, link joints (J0 ~ J6), the TE25W scanning end-effector, the dual-axis turntable’s J0/J1 rotation unit, and the workpiece to be scanned. The diagonal elements in the matrix serve as self-matching identifiers and have no significance for collision detection. Red indicates that the motion spaces of two units overlap, meaning there is a risk of collision. A checkmark indicates that the motion spaces of two units do not interfere with each other, and the units are in a safe operating state. Gray markers indicate the static positional relationships of fixed-mount units; such units have no relative motion, and structural interference can be avoided simply by ensuring initial installation accuracy.

The collision analysis results indicate that there is a risk of collision in the adjacent joint link segments J1-J2, J2-J3, J3-J4, J4-J5, and J5-J6 of the robotic arm. Since the adjacent link segments of the robotic arm are directly connected via the joint axis, when the joint moves to its maximum angular position, structural interference will occur between the link bodies. These areas constitute inherent collision zones that must be prioritized for avoidance as part of the joint motion limit constraints. There is a risk of interference between the robot’s fixed base and the robotic arm’s J0 joint, the turntable’s J0 unit, and the product under test. This issue primarily stems from the limited installation space around the base; when the robotic arm’s root joint rotates significantly, or the turntable rotates to a specific angle, it is prone to scraping against the base and workpieces at fixed stations, thereby imposing boundary constraints on the workspace. There is a risk of collision between the J6 joint at the end of the robotic arm and the TE25W scanning end-effector. This indicates that, during the scanning operation, the end-effector’s working space partially overlaps with the turntable mounting base and the workpiece mounting area, making this a high-risk zone that requires special attention for obstacle avoidance during the path planning phase. The J0 rotary stage of the dual-axis turntable interferes only with the base and the workpiece. The J1 follow-up rotary unit, aside from potential collisions with the J0 unit itself, does not interfere with most of the robotic arm’s joints or the scanning equipment. This indicates that the motion space of the turntable’s J1 axis is well-compatible with the robotic arm’s operational space, and the measurement range can be effectively expanded through the coordinated motion of the robotic arm and the turntable.

**Figure 10:**
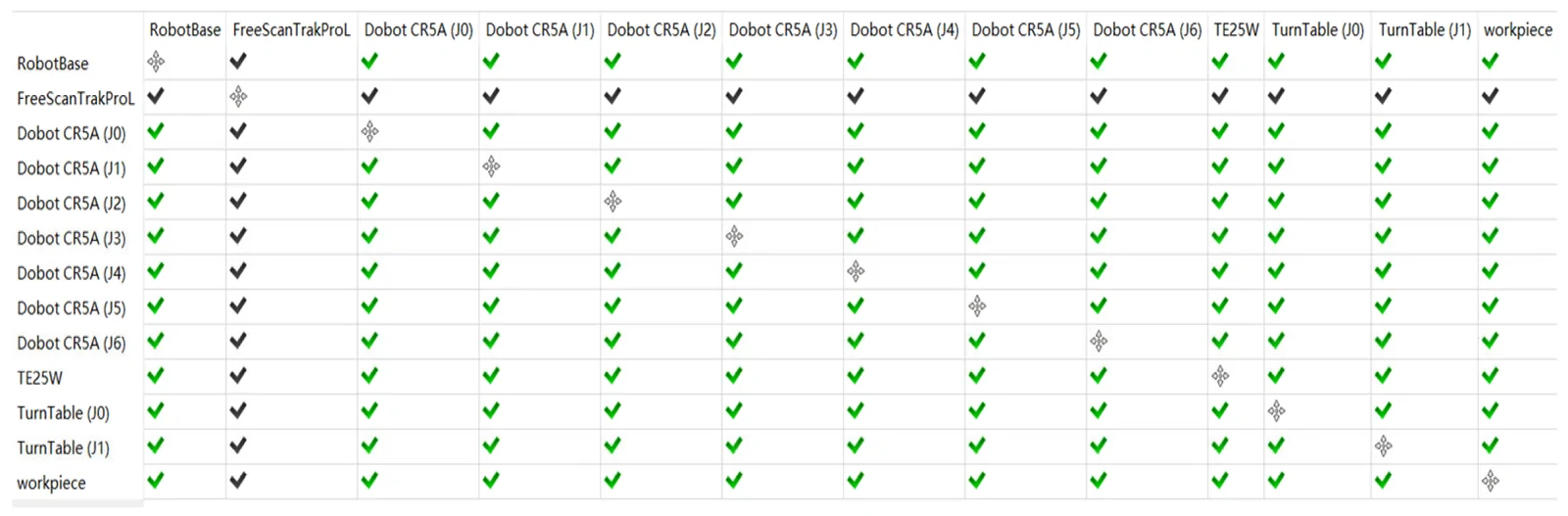
Collision-free robot arm collision simulation map.

Precise minimum distance calculations are performed for potential interference zones, and a safety distance threshold is set. The system traverses the entire trajectory to collect collision data in real time and mark risky locations. By iteratively correcting the trajectory’s orientation and path, the system verifies the trajectory’s safety. Finally, collision simulation analysis yields a system collision detection map, as shown in Figure 10. The analysis results indicate that the system operates without any collision risks. The collision simulation analysis in Figure 9 can effectively prevent collisions between the equipment and the workpiece under test in advance. The measurement trajectory is modified and refined according to the collision analysis. After optimization, a collision-free path, as shown in Figure 10, can be obtained, which can be used for subsequent physical measurement. The measurement environment is equipped with safety guards to prevent personnel intrusion that may interfere with on-site measurement, so as to effectively avoid accidental collisions. In addition, an emergency stop function is configured for the robotic arm. The robotic arm will stop automatically if an unexpected collision occurs. Multi-layer safety protection, including collision simulation analysis, safety guarding, and collision emergency stop, is adopted to prevent safety accidents.

**Figure 11:**
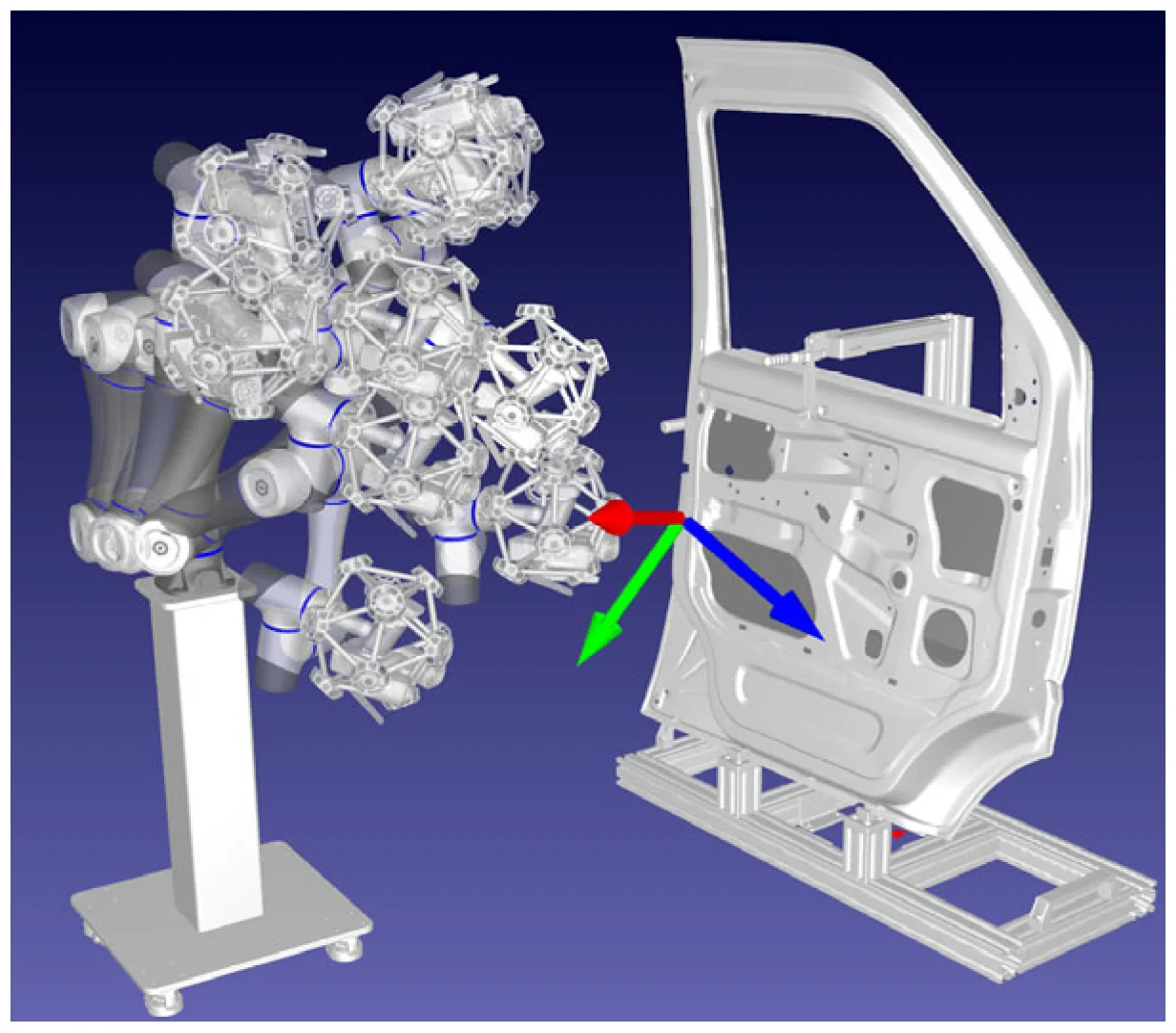
Collision-free robot arm trajectories.

During the product measurement process, a 3D scanning device is mounted on the end-effector of the robotic arm. The product must be scanned and measured without any collisions. Figure 11 shows the 3D scanning trajectory generated after collision analysis. The motion trajectory shows the continuous sequence of movements of the robotic arm throughout the entire scanning operation, clearly illustrating the complete operational envelope of the robotic arm from the base to the end-effector. Judging from the overall shape of the trajectory, the movement of the robotic arm’s joints is smooth and continuous, with no singular configurations or abrupt changes in joint angles. The end-of-arm scanning device consistently advances steadily along the product’s normal, meeting the process parameter requirements for 3D scanning. This trajectory was optimized based on the previously identified collision risks, effectively avoiding potential collision zones and achieving zero interference throughout the operation. If the scanner is too close to the workpiece under test, collision risks tend to occur; if the distance is excessively large, the measurement accuracy will deteriorate. In this work, the distance between the scanning device and the workpiece is generally set to 10–15 cm, which can effectively avoid the above-mentioned issues.

The results show that the trajectory planning method adopted can effectively meet the system’s motion safety requirements while ensuring scanning coverage and pose accuracy, thereby providing a reliable motion plan for the automated 3D inspection of complex structural components. The trajectory simulation process is shown in Table 2.

### 3.3. Key Parameters for 3D Visual Measurement

FreeScan Trak ProL is a wireless, long-range, tracking-based 3D laser scanning system intended for 3D dimensional measurement of large-scale industrial components. This measurement system provides favorable repeatability and high inspection efficiency, together with fast scanning speed and flexible operation. It is widely adopted in automotive, aerospace, construction machinery, energy and heavy industries for dimensional inspection, reverse engineering, and quality control of large-scale components.

**Figure 12:**
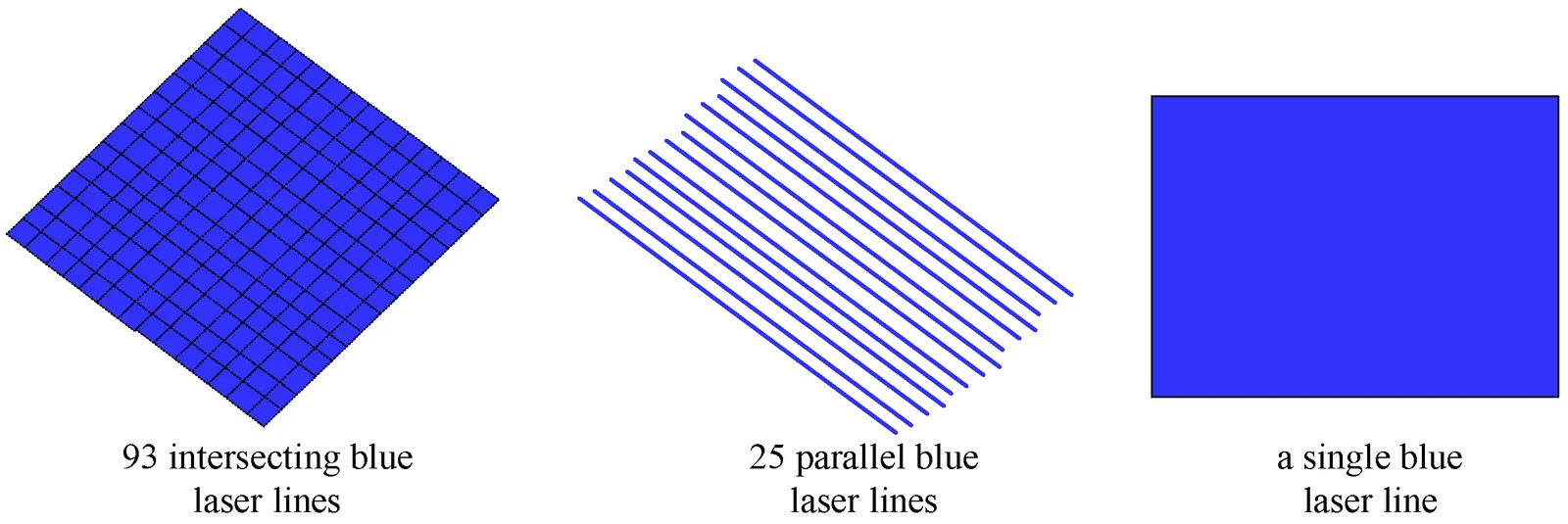
Different laser line-scan modes.

In the automated inspection system designed in this paper, the 3D laser scanning system features a multi-level laser beam architecture that integrates three scanning modes: 93 intersecting blue laser beams, 25 parallel blue laser beams, and a single blue laser beam, as shown in Figure 12. Among these, 93 intersecting laser beams form a grid-like projection to achieve extensive spatial coverage of the surface being measured, enabling efficient acquisition of point cloud data for the workpiece’s overall shape and improving the efficiency of global contour measurement for large-sized components. Twenty-five parallel laser beams form an equidistant linear array projection to achieve high-density sampling of localized areas, accurately reproducing minute geometric features and complex surface topography to meet the high-precision inspection requirements for fine features. Single-beam lasers are designed for shallow, optically accessible recessed features only. Direct optical scanning is infeasible for deep enclosed holes and fully enclosed internal cavities due to line-of-sight occlusion. For shallow recessed regions that maintain clear optical access, the directed laser beam acquires 3D topography data and extends measurable outer-surface boundaries. No measurement data can be obtained for fully enclosed internal cavities. Flexible switching between modes allows the automated inspection system to adjust its scanning strategy based on the structural characteristics of the object being measured, achieving a balance between measurement efficiency and detail resolution.

The automated scanning system described in this paper demonstrates outstanding industrial-grade performance in terms of accuracy, resolution, and data acquisition efficiency. According to the equipment specification, the integrated photogrammetry system provides a volumetric accuracy of 0.044 mm + 0.012 mm/m, which serves as the hardware index for the scanner and meets the inspection requirements for typical industrial components. The spatial resolution is adjustable within a range of 0.01–10 mm, allowing for flexible control of point cloud density based on inspection requirements. This balances the detailed characterization of minute features with the efficient modeling of large-scale structures. The scanner hardware supports a theoretical maximum point cloud acquisition rate of 7.6 × 10^6^ points per second. This value represents the peak hardware capacity of the device. Actual effective point-acquisition speed under real inspection conditions is lower and depends on workpiece geometry, scanning distance, and adopted laser operating mode, as reflected in the experimentally measured inspection cycle times presented in Section 4.2.

### 3.4. Automated Inspection System

**Figure 13:**
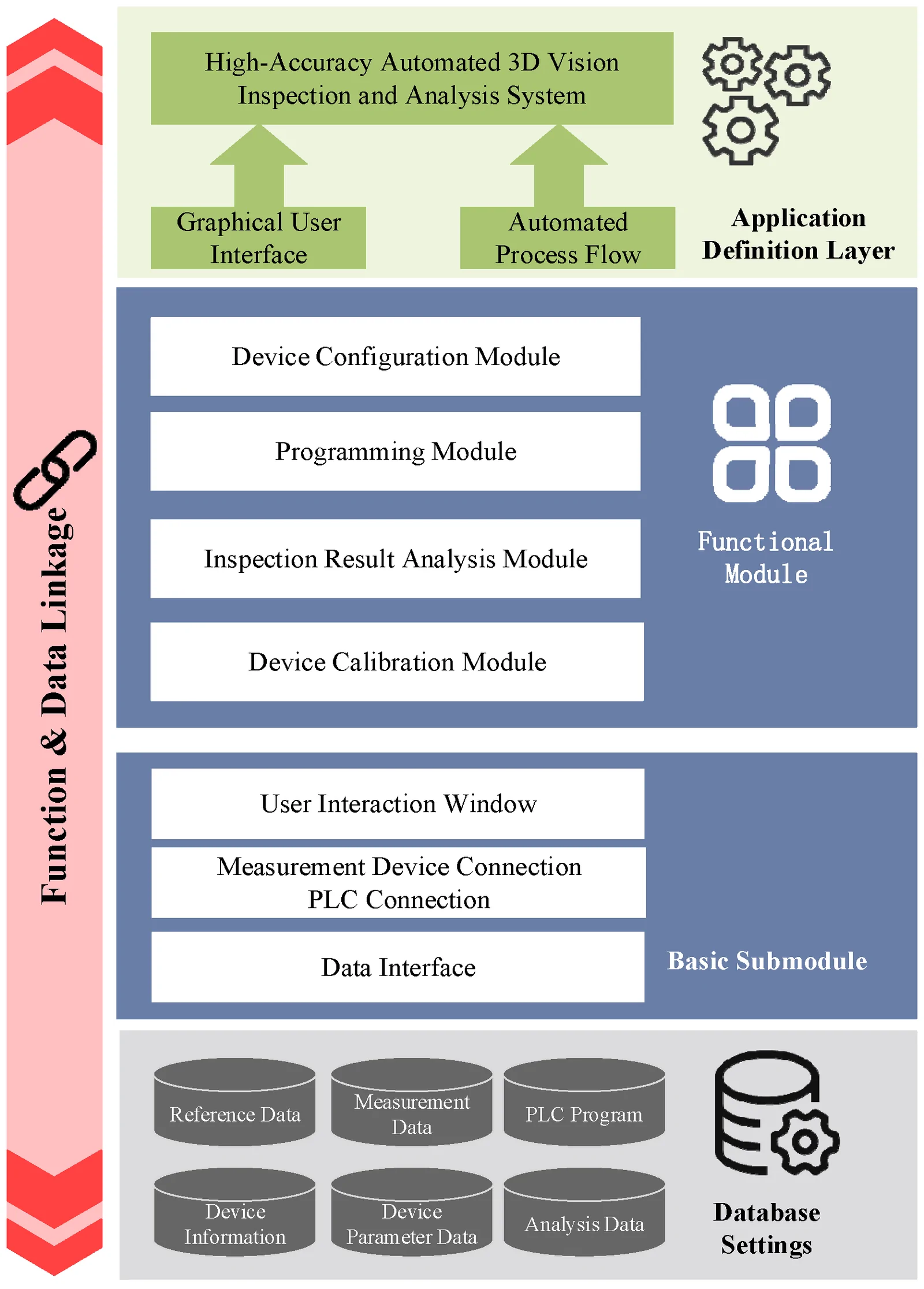
Software framework for a high-precision 3d vision-based automated inspection and analysis system.

The high-precision 3D vision-based automated inspection and analysis system proposed in this paper adopts a hierarchical, modular architectural design approach. It is organized from the bottom up into four layers: the data layer, the basic module layer, the core functional module layer, and the application layer. Each layer achieves information coordination through module functionality and data interaction, ensuring both the system’s functional integrity and good scalability and maintainability. The software system framework is shown in Figure 13.

The underlying architecture of the automated inspection software described in this paper consists of a database configuration layer that centrally manages business data throughout the entire process. The reference data module is primarily used to store standard numerical models, benchmark data, and other criteria for inspection and evaluation. The measurement data module manages acquired results such as 3D point clouds and raw scan signals. The PLC module stores the motion control logic and scheduling programs for the automated production line. The Equipment Information module records the basic attributes of hardware such as scanning devices and motion actuators. The Equipment Parameters module stores parameters such as calibration settings and scan accuracy configurations. The Analysis Data module primarily stores data such as deviation analysis reports, defect statistics, and quality assessments. A unified data storage and management mechanism ensures the traceability and reusability of data throughout the entire automated inspection process, providing stable data support for automated measurement.

The software base module serves as the hub connecting the hardware system to higher-level functions, encapsulating the underlying communication and interaction logic. This layer includes a user interface window that provides operators with a visual operating environment. The measurement device connection and PLC connection modules enable communication between 3D scanning equipment, programmable logic controllers, and the software, supporting real-time monitoring of hardware status, the issuance of motion commands, and the transmission of acquired data. The data interface unit provides standardized data import and export protocols, enabling data exchange between the system and production management systems as well as third-party analysis software.

The core functional module layer of the software serves as the central execution unit for the system’s inspection operations. The device configuration module is used to configure scanning parameters, motion parameters, and the hardware configuration of inspection stations, as well as to debug parameters. The programming module supports inspection path planning, scanning process setup, and offline programming and motion simulation of automated inspection programs. The inspection results analysis module is used for core analytical functions such as point cloud data processing, 3D reconstruction, deviation calculation, defect identification, and inspection report generation. The equipment calibration module provides functions such as system hand–eye calibration, accuracy calibration, and multi-coordinate system registration analysis, thereby ensuring the stability and reliability of automated inspection. The software application layer is designed for end users and industrial applications. Through graphical user interfaces and automated workflows, this layer ultimately achieves the system goal of high-precision automated 3D vision inspection and analysis. The software’s overall architecture features strong decoupling between modules, allowing for flexible expansion of functional units based on different inspection objects and application scenarios to meet the diverse 3D vision automation inspection needs of industrial settings.

**Figure 14:**
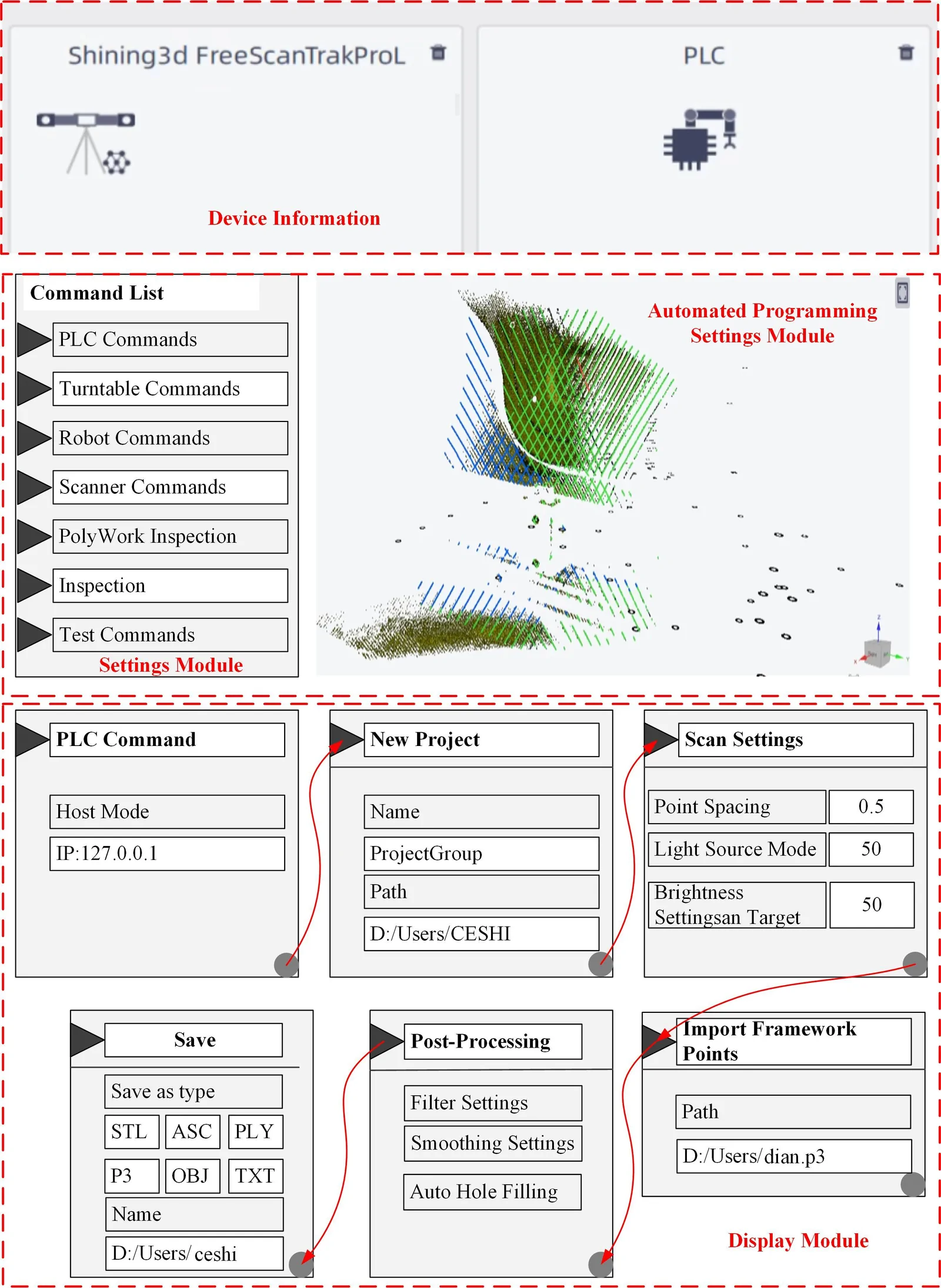
High-precision 3D vision automation inspection and analysis software.

The high-precision 3D vision-based automated inspection and analysis software proposed in this paper employs a modular execution logic that covers equipment management, project configuration, path planning, automatic data acquisition, data processing, and result output, thereby forming a complete closed-loop automated inspection system. The overall execution flow of the software is shown in Figure 14. The software establishes communication links between the 3D scanning unit and PLC control system via its equipment-information module. A unified command list encapsulates control instructions for the PLC, fixture unit, robotic arm, and scanner, enabling coordinated scheduling of hardware resources and performing system initialization and pre-inspection hardware calibration.

The software includes a project management module for creating inspection tasks and defining data storage directories. Users configure key acquisition parameters, including point cloud resolution and light source settings within the parameter panel. Reference point data defined in the workpiece coordinate frame are imported to build the inspection coordinate system, completing initial parameter setup and fiducial registration for each measurement task. The software sequentially issues motion control and data acquisition commands according to the preset inspection program, driving the robotic arm, fixture turntable, and scanning equipment to work in coordination. It automatically completes the acquisition of 3D point cloud data from the surface of the product under test. Throughout the process, the software synchronizes equipment operating status and acquisition progress in real time to ensure the automation and stability of the inspection process. After measurement is complete, the software performs standard post-processing on the point cloud data, including filtering, smoothing, and hole repair. Among them, the filtering level can be set to low, medium, or high. The smoothing function provides standard, medium, and high modes. For hole-filling operations, the hole-perimeter threshold can be user-customized. A perimeter value of 10 was adopted throughout our experiments. This hole-filling reconstruction is applied exclusively to small local holes generated where optical target markers obscure the outer workpiece surface. Importantly, these computationally reconstructed filled regions are strictly excluded from all subsequent deviation-analysis workflows. Only point-cloud points originating from direct laser scanning of physical workpiece surfaces are used for geometric deviation and topographic error computation. Reconstructed hole-filled patches do not contribute to inspection metrics, and this reconstruction function cannot recover geometry information for deep enclosed internal cavities. It then uses a reference model to conduct inspection and analysis of deviations and topographic errors. Finally, the software supports data export in multiple common formats and project saving, enabling traceable management of inspection results. The software system proposed in this paper achieves full-process automated control of 3D visual inspection, effectively improving the efficiency and standardization of automated inspection.

## 4. Validation of Automated Inspection Systems

### 4.1. Automated Inspection Platform

**Figure 15:**
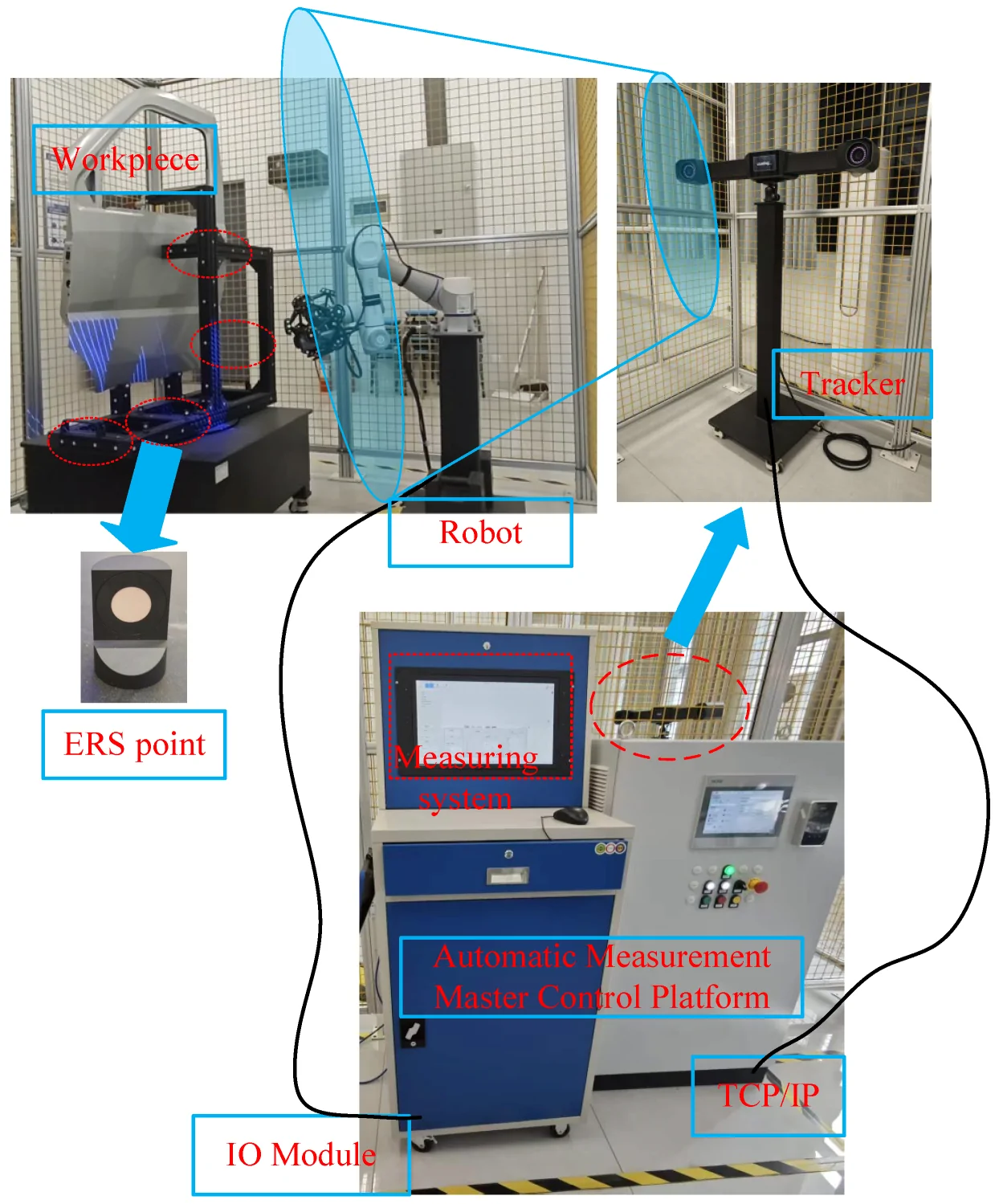
Automated inspection platform.

This paper describes the development of a high-precision 3D vision-based automated inspection platform. The system’s hardware and software components primarily include an industrial robot (Dobot CR5A), a 3D scanner (FreeScan Trak ProL), reference points, a measurement and analysis system, and a central control platform. The overall system configuration is shown in Figure 15. Among them, for the FreeScan Trak ProL scanner used in experiments, the point spacing was set to 0.5 mm, the light-source operating mode was configured as mixed multi-laser mode, the light-source intensity parameter was set to 50, and the target-marker brightness setting was set to 50. The ERS point layout was arranged to cover the entire structure according to the fixture geometry, ensuring that the device can capture the points after the fixture rotates. The central control platform serves as the top-level coordination hub, overseeing the robot’s motion control, 3D scan data acquisition, and the tracker’s position and orientation monitoring. The ERS points serve as a global coordinate reference, unifying the coordinate systems of all measurement devices to enable the automated, high-precision acquisition, inspection, and analysis of the 3D contour information of the workpiece under test.

### 4.2. Experimental Analysis

**Figure 16:**
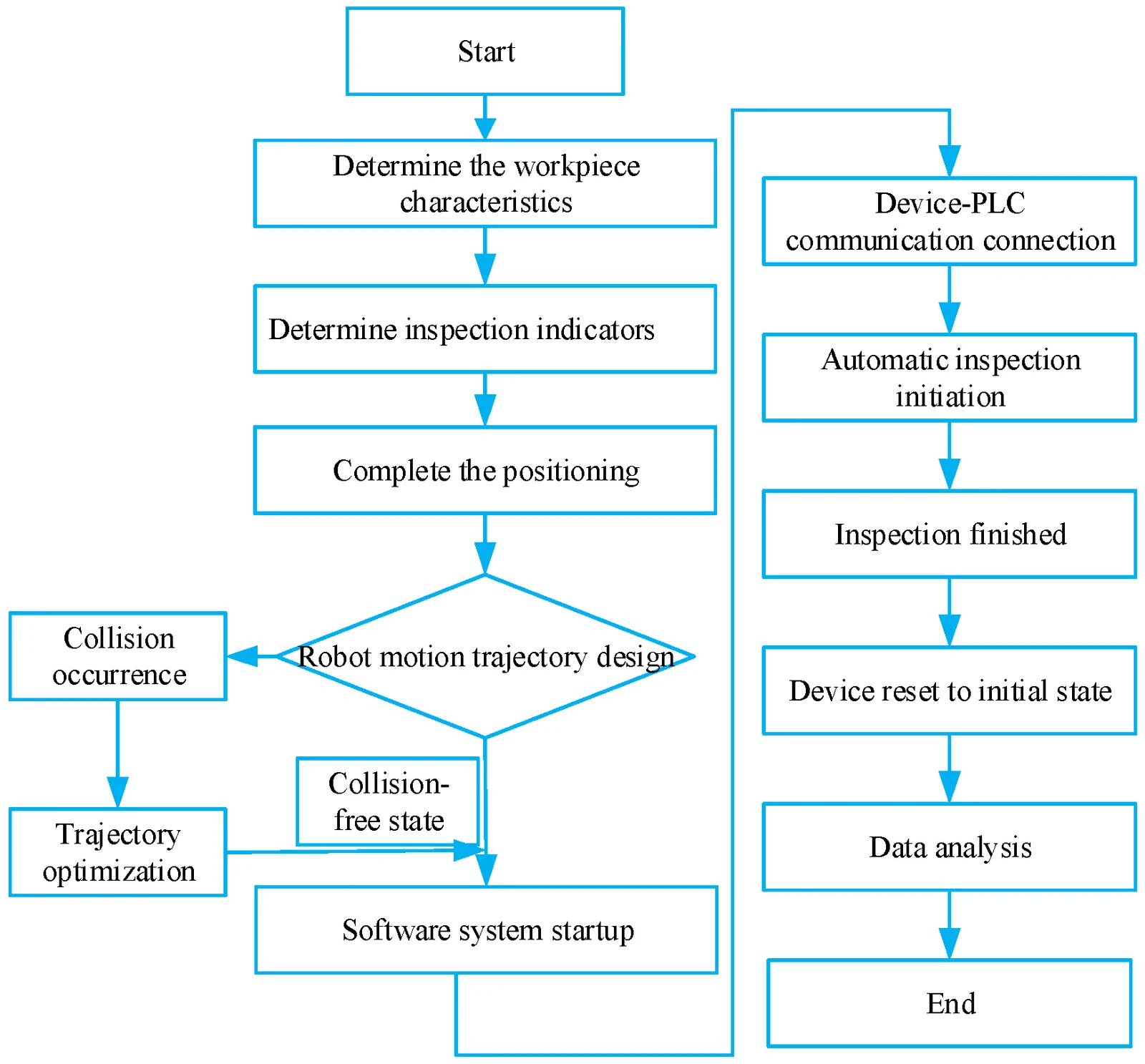
System operation process.

First, based on the structural characteristics and specification requirements of the workpiece under test, the workpiece is precisely clamped into the fixture and positioned and secured. By combining the workpiece’s outer contour with the principles of inspection path planning, the robot’s spatial motion trajectory is designed and generated, and a collision analysis of the measurement end-effector is performed to proactively mitigate the risk of collisions between the equipment, the workpiece, and the fixture during the inspection process. Next, the automated inspection and analysis software system is launched to establish communication and configure parameter matching among the measurement unit, PLC control system, and main control platform. Finally, by issuing operation commands via the industrial control terminal, the automated inspection platform enters a fully automatic operating state. Once all inspection procedures are completed, the robot automatically returns to its initial origin, and the fixture rotates back to its initial orientation. The acquired 3D point cloud data is filtered, registered, and processed by the analysis software to complete the workpiece surface quality analysis and the evaluation of geometric feature parameters, and a standardized inspection report is generated. The overall system operation process is shown in Figure 16.

**Figure 17:**
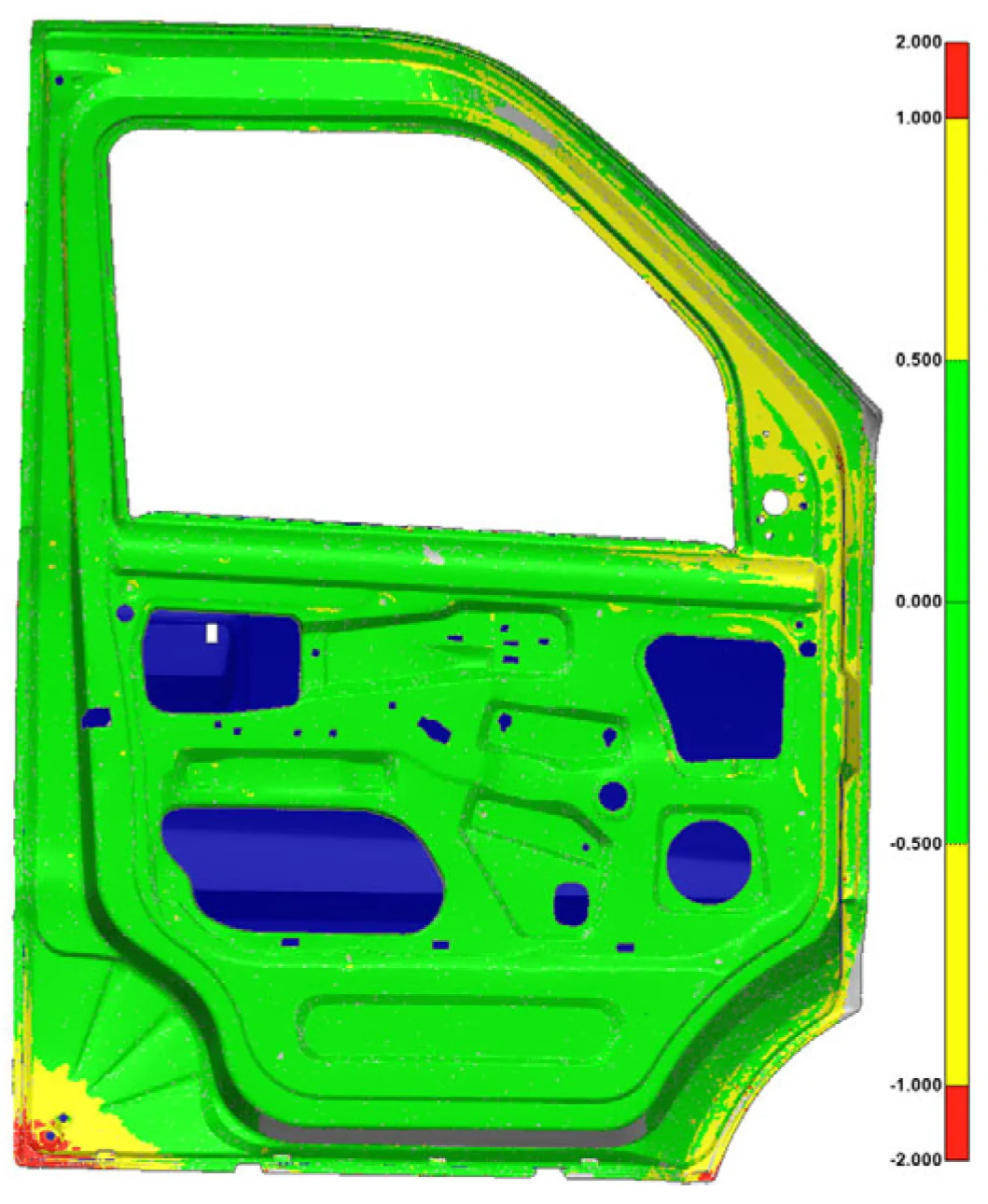
Analysis of surface profile deviations in car doors.

To verify the detection performance and engineering applicability of the high-precision 3D vision-based automated inspection platform developed in this paper, an automobile door was selected as a typical test object for validation testing. The door was clamped and secured in the rotary fixture designed in this paper; after positioning and calibration were completed, the fully automated inspection process was initiated to acquire complete 3D topographic data of the door. Next, after precisely registering the measured point cloud with the standard digital model, the analysis results for the door surface contour deviations were output, as shown in Figure 17.

As shown in the deviation cloud map, the main body surface of the car door is predominantly displayed in green, with surface contour deviations generally falling within the ±0.5 mm range. This indicates good forming accuracy of the workpiece’s main surface and demonstrates the inspection system’s stability in measuring large, complex surfaces. Yellow and red deviation zones are distributed along the door edges, corners, and localized protrusions, with maximum deviation values of approximately ±2.0 mm. These deviations are primarily caused by process factors such as material springback and flanging errors during the stamping process. The analysis results indicate that the system described in this paper can accurately identify areas where the workpiece’s dimensions exceed tolerance limits.

The overall test results indicate that the automated inspection platform developed in this paper can efficiently inspect complex-surface workpieces such as automotive doors. It provides a visual representation of the distribution patterns and local characteristics of surface contour deviations, offering data support for workpiece quality assessment and process improvement, thereby validating the system’s inspection capability and engineering value. It should be noted that three categories of regions appear within the inspection results. The majority of point cloud data originates from direct laser scanning of physically exposed workpiece surfaces and participates in deviation calculation. Small local holes caused by attached optical markers are computationally reconstructed via hole-filling operations. These reconstructed regions are excluded from deviation analysis. Surfaces blocked by clamping hardware or deep enclosed cavity geometries represent optically inaccessible zones, for which no valid measurement data can be obtained. Note that deviations between measured point cloud and nominal CAD geometry combine workpiece manufacturing geometric variations together with measurement-related error. Such CAD-model comparison cannot serve as metrological proof for the dimensional measurement accuracy of the inspection system.

**Figure 18:**
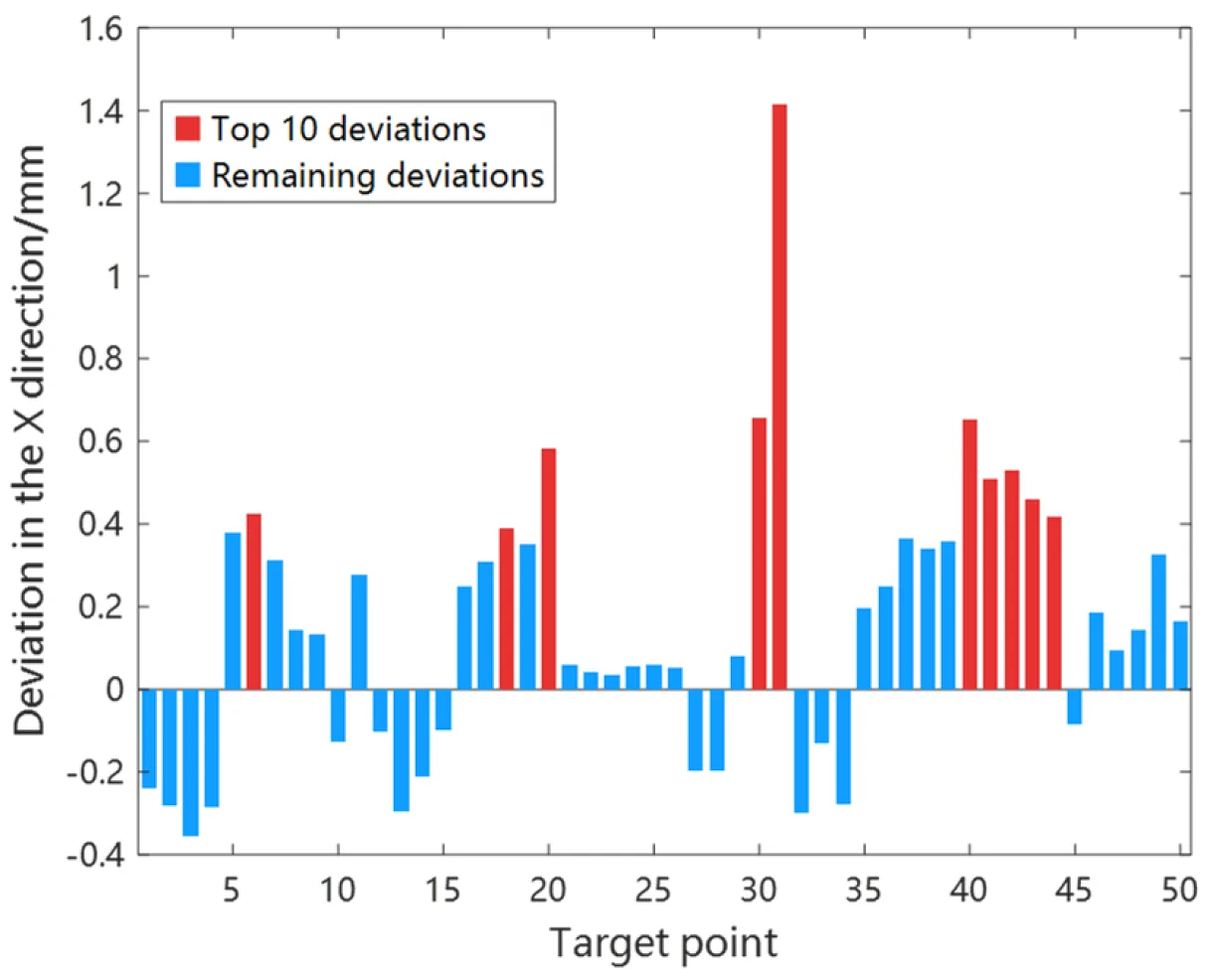
X-direction deviation analysis.

**Figure 19:**
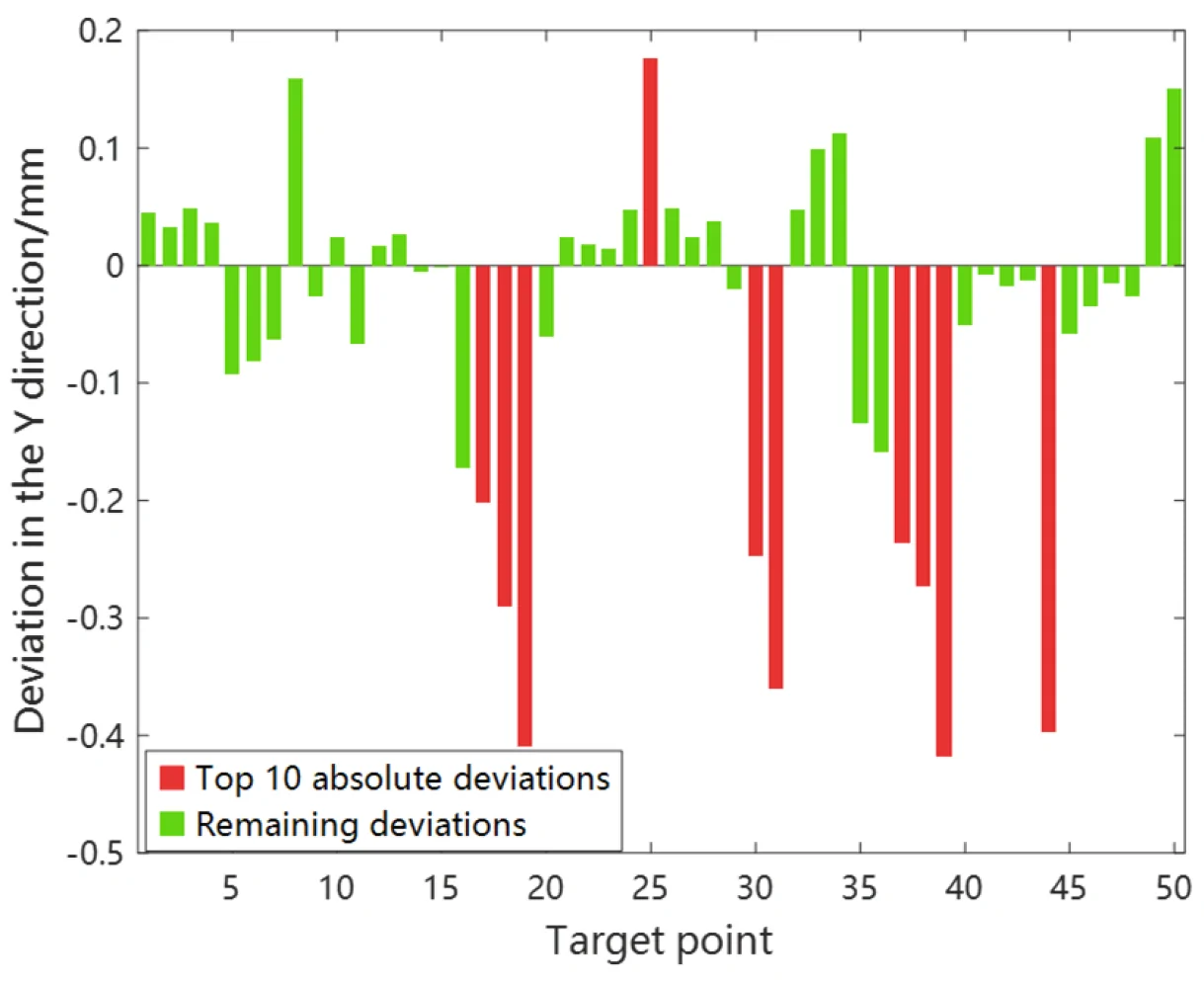
Y-direction deviation analysis.

**Figure 20:**
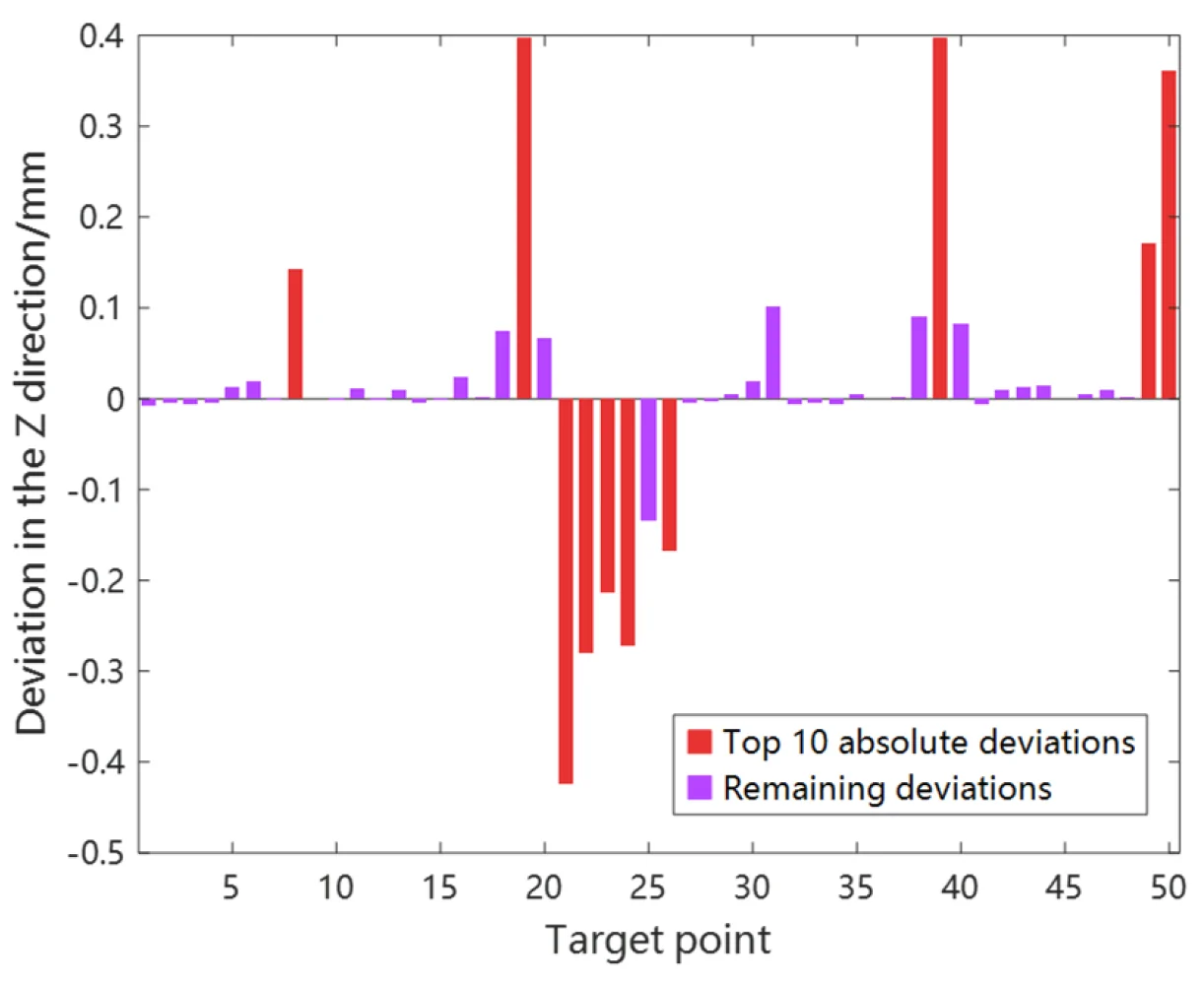
Z-direction deviation analysis.

To analyze the measurement results more accurately, 50 measurement points were randomly selected on the car door. The deviation results in the X, Y, and Z directions are shown in Figure 18, Figure 19 and Figure 20. The measurement deviations in each direction exhibit significant directional differences. The deviations in the X direction are generally the largest; among the top 10 measurement points with the greatest deviations, the maximum positive deviation reaches 1.41 mm, while the deviations at the remaining measurement points are mostly within the ±0.4 mm range, with positive and negative deviations alternating. Among the top 10 measurement points with the largest deviations in the Y direction, the deviations were predominantly negative, with a maximum absolute value of approximately 0.39 mm. The deviations at the remaining measurement points were relatively small, generally falling within the range of ±0.15 mm. Among the top 10 measurement points with the largest deviations in the Z direction, the deviations were close to 0.39 mm. In contrast, those at the remaining measurement points were close to zero, indicating good measurement stability.

Deviations in the *x*, *y*, and *z* directions better illustrate the contribution of each measurement point to the error in a specific direction. To comprehensively evaluate the deviation status of measurement points, the total deviation method is used to assess measurement quality.

(7) $$T_{d}=\sqrt{{\left(\mathrm{dx}\right)}^{2}+{\left(\mathrm{dy}\right)}^{2}+{\left(\mathrm{dz}\right)}^{2}}$$

where, *dx*, *dy*, and *dz* represent the measurement deviations in the *x*, *y*, and *z* directions, respectively.

**Figure 21:**
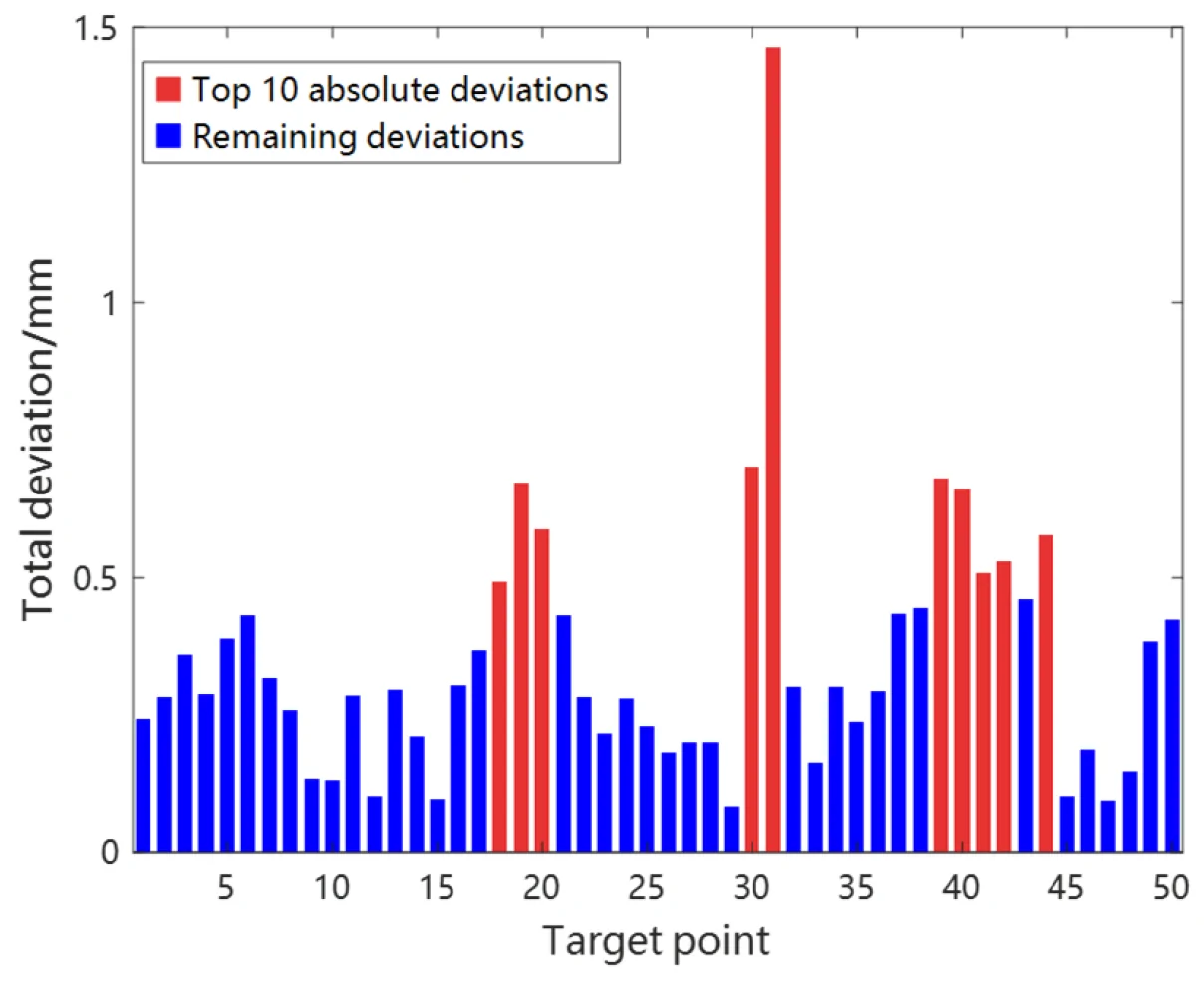
Overall deviation analysis.

Owing to the large volume of point cloud data acquired from scanning measurements, plotting and displaying deviation results for the full point cloud is impractical. Therefore, to intuitively visualize deviations of measurement points, 50 measurement points were selected in this study. The custom software analysis system was adopted to identify the top ten points with the largest deviations for rapid localization, as illustrated in Figure 21. The developed software system allows users to customize the number of measurement points for deviation analysis according to practical requirements. It also supports deviation sorting and extraction of points with significant deviations at a specified proportion, improving the visualization of inspection results and the flexibility of data analysis.

Deviations between the measured point cloud and the nominal CAD model mainly reflect the geometric variations of the workpiece itself, rather than the absolute measurement accuracy of the inspection system. To characterize the performance of the proposed platform, we supplemented repeatability experiments using fixed marked points. Twenty repeated measurements were carried out at predefined positions, and measurement uncertainty was evaluated to quantify the repeatability of the automated inspection platform. Meanwhile, we compared the results obtained by automated inspection and manual reference measurement to further verify the platform performance.

The marker-based tests described below quantify point-coordinate repeatability. This refers to the reproducibility of the three-dimensional coordinates of discrete fiducial markers across repeated measurement cycles, as distinct from the repeatability of the complete surface-scanning workflow. During these tests, the platform operates in single-point coordinate measurement mode and records only the coordinates of the attached target markers. Accordingly, dense point-cloud acquisition, mesh reconstruction, and deviation computation are not involved in this evaluation.

**Figure 22:**
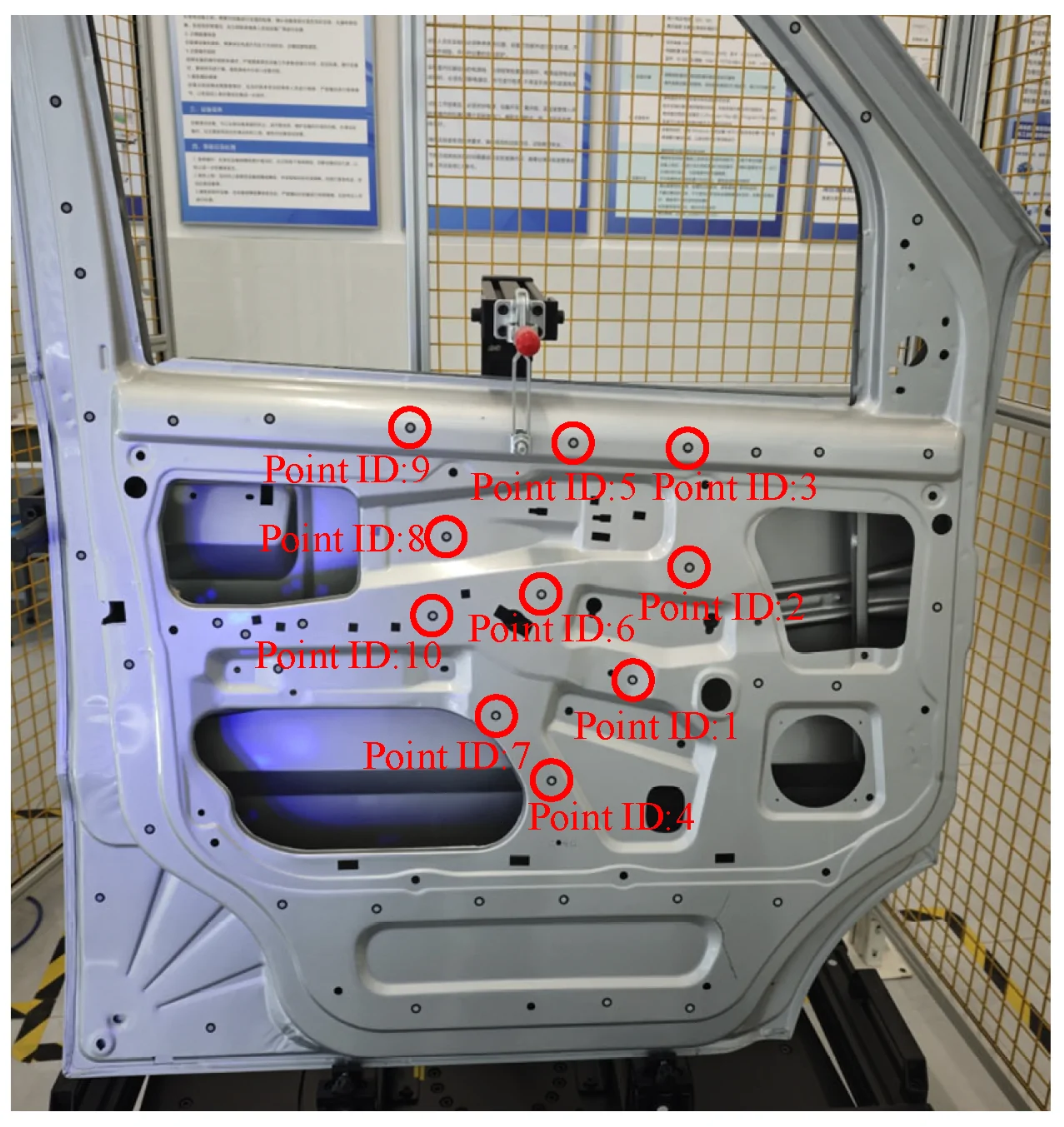
Schematic diagram of measurement point selection.

To further evaluate the impact of the automated measurement method proposed in this paper on the repeatability of measurement points during the actual measurement process, 10 fixed measurement points, as shown in Figure 22, were selected. Using the experimental platform developed in this paper, 20 measurements were taken at 10 fixed locations. Through multiple automated measurements, we evaluated the impact of the automated measurement platform described in this paper on the repeatability of measurements at each point. The 20 repeated measurements were implemented as independent full measurement cycles. The workpiece was kept mounted on the rotatable fixture without re-clamping or disassembly during all repeated tests. Coordinate registration was renewed in each measurement run, and the fixture was rotated to the predefined fixed angle for data collection. High-precision target markers were affixed on the door surface as fixed reference points with predefined physical locations. During the repeatability test, the system was operated under single-point coordinate measurement mode instead of scanning mode. Dense point cloud data was not collected; only the 3D coordinates of these target markers were recorded. The 3D coordinates of predefined targets were extracted in each measurement cycle to consistently track the identical physical points across multiple measurement trials. Under stable laboratory environmental conditions and within the short test duration, no obvious measurement drift was observed for this set of experiments.

The initial coordinates of the 10 fixed points shown in Figure 22 are listed in Table 3. To evaluate the repeatability of the automated measurement platform described in this paper, Class A standard uncertainty was used to assess the accuracy of the measurement points.

The measured point coordinates are as follows:

(8) $$P^{M}=\left\{P_{1}^{M},P_{2}^{M},P_{3}^{M},\ldots ,P_{n}^{M}\right\}$$

(9) $$P_{i}^{M}=\left(x_{i},y_{i},z_{i}\right)$$

The deviations of each component in a single measurement relative to the mean value of the measurement points in this group are:

(10) $$dx_{i}=x_{i}-\bar{x}$$

(11) $$dy_{i}=y_{i}-\bar{y}$$

(12) $$dz_{i}=z_{i}-\bar{z}$$

The sample standard deviations for each directional component are:

(13) $$s_{x}=\sqrt{\frac{1}{n-1}\sum_{i=1}^{n}{\left(dx_{i}\right)}^{2}}$$

(14) $$s_{y}=\sqrt{\frac{1}{n-1}\sum_{i=1}^{n}{\left(dy_{i}\right)}^{2}}$$

(15) $$s_{z}=\sqrt{\frac{1}{n-1}\sum_{i=1}^{n}{\left(dz_{i}\right)}^{2}}$$

The measurement uncertainty for each directional component is expressed as:

(16) $$u_{x}=\sqrt{\frac{1}{n\left(n-1\right)}\sum_{i=1}^{n}{\left(dx_{i}\right)}^{2}}$$

(17) $$u_{y}=\sqrt{\frac{1}{n\left(n-1\right)}\sum_{i=1}^{n}{\left(dy_{i}\right)}^{2}}$$

(18) $$u_{z}=\sqrt{\frac{1}{n\left(n-1\right)}\sum_{i=1}^{n}{\left(dz_{i}\right)}^{2}}$$

The spatial deviation of the measurement points is:

(19) $$d_{i}=\sqrt{{\left(dx_{i}\right)}^{2}+{\left(dy_{i}\right)}^{2}+{\left(dz_{i}\right)}^{2}}$$

The expression calculates the standard uncertainty of the mean radial deviation.

(20) $$u_{d}=\sqrt{\frac{1}{n\left(n-1\right)}\sum_{i=1}^{n}{\left(d_{i}-\bar{d}\right)}^{2}}$$

where *n* denotes the 20 repeated measurements acquired at each fixed point. *d_i_* is the Euclidean distance between the measurement points, and $\bar{d}$ is the average of the Euclidean distances between the measurement points. This expression yields the standard uncertainty of the mean radial deviation obtained from repeated point-coordinate measurements (Type-A evaluation). This quantity only reflects point-measurement repeatability under fixed clamping conditions and does not represent a full combined measurement uncertainty budget. Additional uncertainty sources including registration error and fixture positioning error are not incorporated in this calculation.

**Figure 23:**
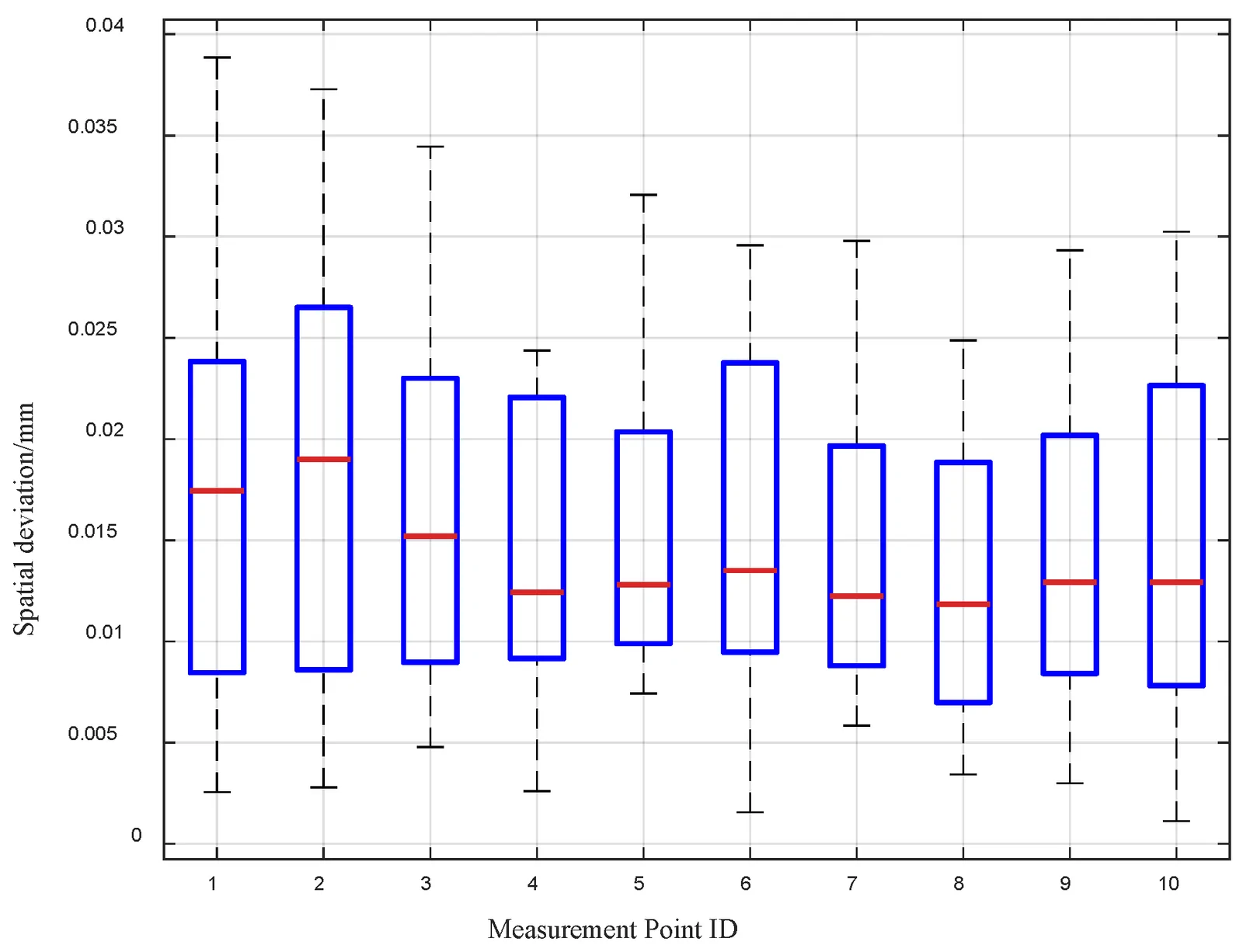
Measurement point spatial deviation.

Figure 23 shows a box-and-whisker plot of spatial deviations obtained from 20 repeated measurements at fixed measurement points. In the box plots, the red horizontal line represents the median deviation for each set of measurement data, and the dashed line indicates the range of deviations in the measurement data. The results show that the spatial deviation at fixed measurement points falls within the range of 0 to 0.04 mm, indicating a low level of dispersion in repeat measurements overall. This further demonstrates that the automated inspection platform described in this paper possesses excellent point-coordinate repeatability.

As can be seen from the above results, Measurement Point ID. 2 has the largest median spatial deviation, indicating that the repeatability of measurements at this point is relatively poorer than at other points. Measurement Point ID. 8 has the smallest median spatial deviation, indicating the best repeatability at this point. The median spatial deviations of the remaining fixed measurement points are concentrated between 0.012 and 0.019 mm, with relatively little variation overall. Repeatability tests showed that the spatial deviations from repeated measurements at fixed measurement points did not exhibit any significant systematic drift. The measurement deviations were concentrated around the median, confirming that the point-coordinate repeatability of this automated measurement platform meets the inspection requirements.

The standard uncertainties of the measurement points, calculated using Equations (8)–(20), are shown in Table 4, where *u_x_*, *u_y_*, and *u_z_* represent the standard uncertainties of the measurement points along the x, y, and z coordinate axes, respectively, and *u_d_* is the combined standard uncertainty of the radial distance calculated from the three orthogonal coordinate components of the measurement point. The experimental results show that the uncertainty at the measurement point is smaller in the x and z directions but larger in the y direction. Slight directional differences in observed uncertainty originate primarily from random measurement noise. Isolating and quantifying any potential systematic positioning bias requires dedicated calibration experiments with traceable reference artifacts. Such calibration work is reserved for future research. The combined standard uncertainty for the 3D spatial coordinates of the measurement points remains generally low. Slight variations in uncertainty are observed across different measurement points, which can be attributed to factors including measurement-point position and scanning angle. Collectively, the experimental results demonstrate that the automated inspection platform proposed in this work yields low point-coordinate-related uncertainty with stable point-coordinate repeatability performance, satisfying the requirements for practical inspection tasks.

Values in this table are Type-A standard uncertainties calculated from 20-time repeated fixed-point measurements under unchanged clamping conditions. They characterize only point-measurement repeatability and do not constitute full combined measurement uncertainty. Validation with traceable calibrated references, bias assessment, evaluation after workpiece re-clamping and rotation, and complete uncertainty budgeting are left for future investigations. It should be emphasized that all repeatability statistics shown in Figure 23 and Table 4 are acquired under fixed-clamping conditions, where the workpiece remains mounted without disassembly or re-clamping between measurement cycles. These tests only evaluate discrete marker-point repeatability and do not include dense-surface scanning repetition after workpiece re-clamping or fixture re-orientation. Traceable dimensional validation against calibrated reference artifacts, quantitative measurement-bias estimation, and a comprehensive uncertainty budget accounting for all error sources were not performed in the present study and constitute important directions for follow-up research. CAD-model comparison results reported above cannot be used to derive the absolute dimensional metrological accuracy of the inspection platform.

**Figure 24:**
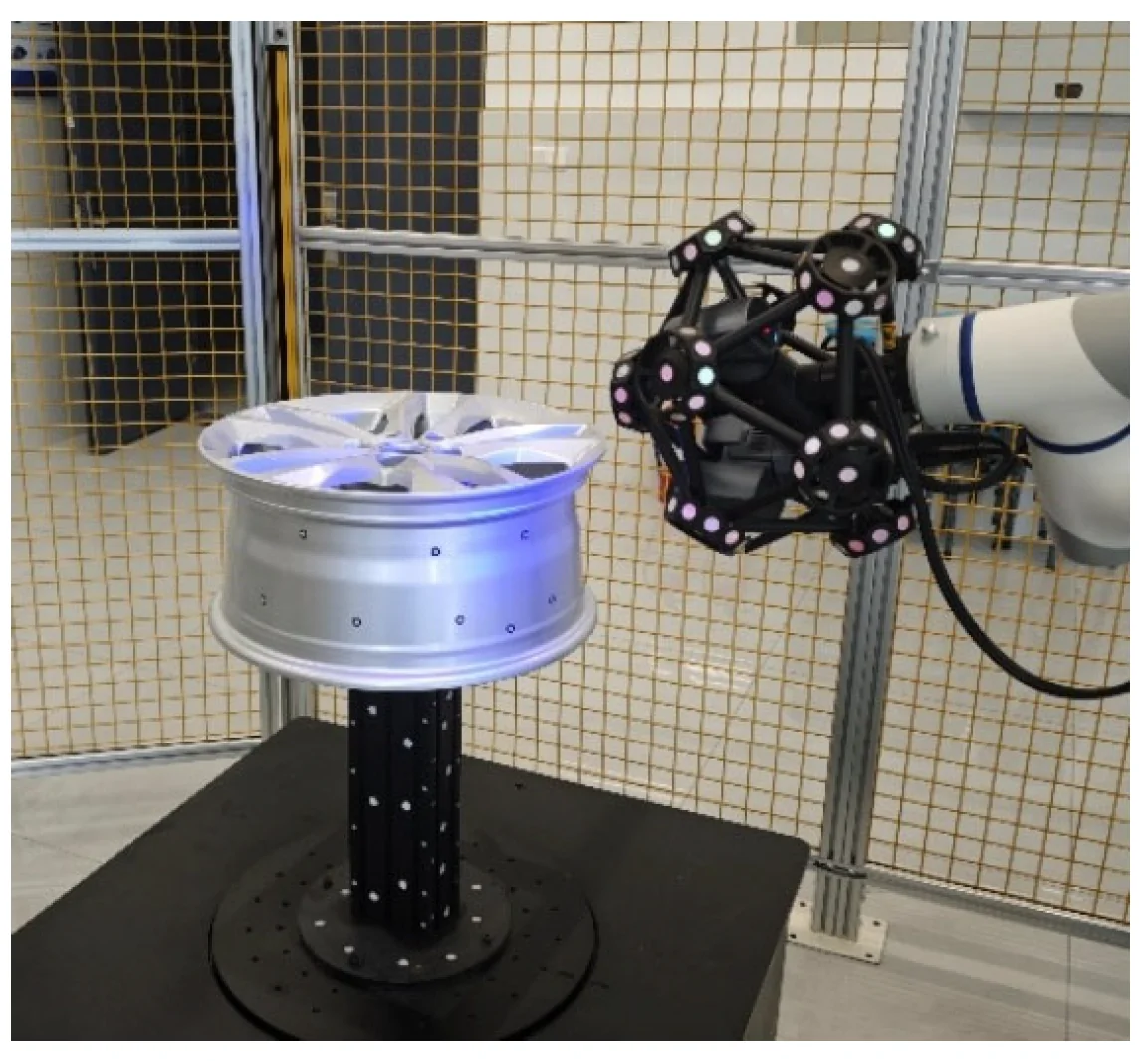
Wheel hub inspection test platform.

To further verify the generalization capability of the proposed automated inspection platform for diverse products, comparative tests between manual and automated measurement approaches were performed on the wheel-hub test setup illustrated in Figure 24.

In contrast to the marker-based tests above, which isolate point-coordinate repeatability, the three repeated automated inspections of the wheel-hub specimen assess the repeatability of the complete surface-scanning workflow, including dense point-cloud acquisition, registration, post-processing, and deviation analysis.

Table 5 compares the inspection cycles for wheel hubs. The comprehensive results show that the total cycle time for manual measurement was 25 min, while the total time required using the automated inspection platform described in this paper was 11 min. A comparison of the overall cycles indicates that the automated inspection cycle is shorter. Manual measurement demands high operator expertise, and variations in measurement habits among different operators may yield inconsistent results. The proposed automated inspection platform adopts pre-planned measurement paths and fixture-based workpiece clamping to reduce human intervention. Inspection templates can be established for identical components to realize batch inspection, substantially boosting inspection efficiency.

Each full inspection cycle starts from the moment the workpiece is securely clamped in place and ends when the deviation analysis report is exported. For manual measurement, no automated fixture rotation is performed; hence, fixture-motion time equals zero. The total cycle time sums scanning, fixture-motion, point-cloud-processing and report-generation durations. Both manual and automated tests were configured with identical predefined surface-coverage targets for the wheel-hub specimen.

**Figure 25:**
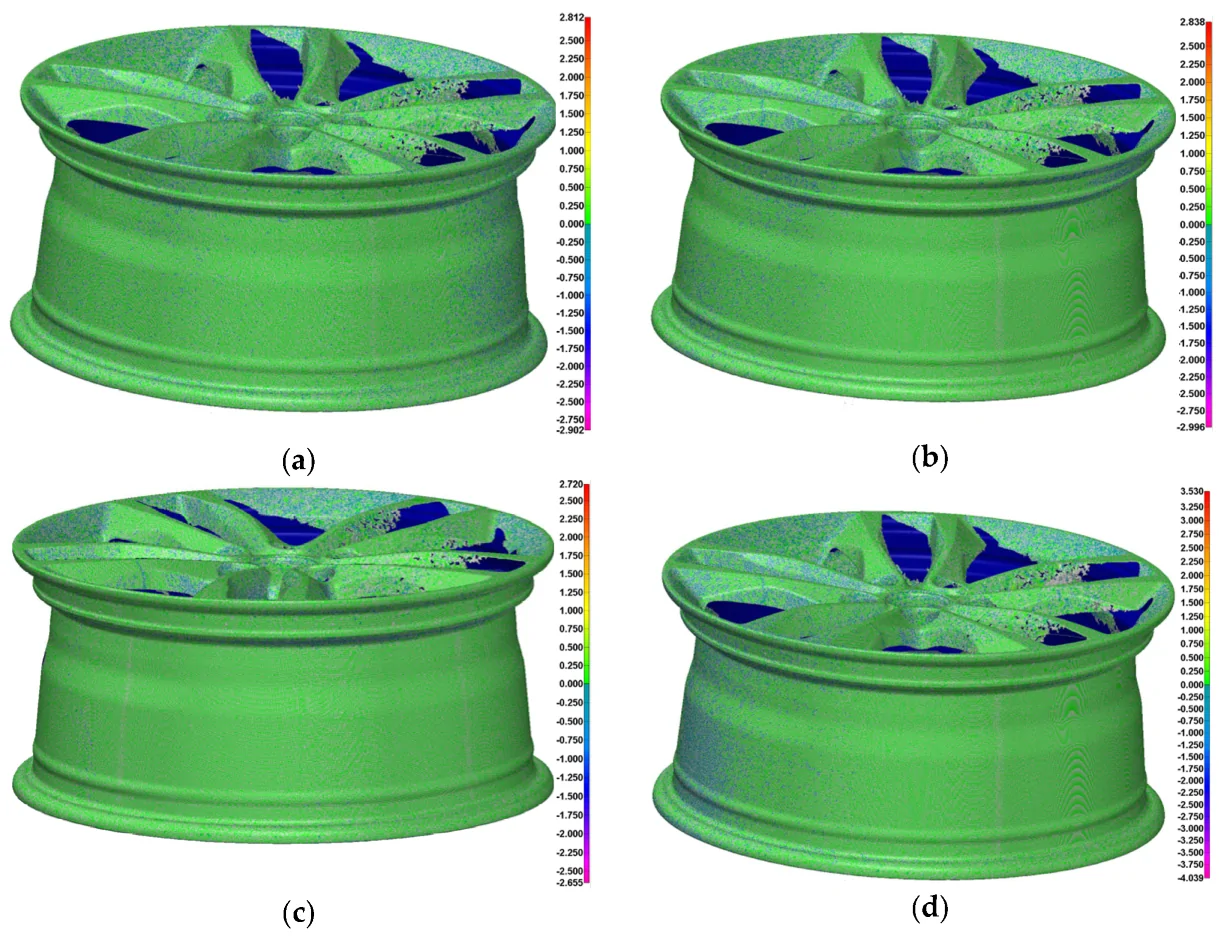
Comparison between manual and automated measurements. (**a**) First automated inspection result of the wheel-hub. (**b**) Second automated inspection result of the wheel-hub. (**c**) Third automated inspection result of the wheel-hub. (**d**) Manual measurement result.

Figure 25 presents the manual and automated measurement results. Figure 25a–c reveal that following three repeated automated inspections on the wheel-hub test specimen, the maximum positive deviations are 2.812 mm, 2.838 mm and 2.720 mm, respectively. The comparable magnitude of maximum deviations across these repeated measurements further validates the repeatability of the proposed automated inspection platform. Figure 25d presents the manual measurement results, with a maximum positive deviation of 3.53 mm. This value is considerably larger than that obtained from automated measurements. Such prominent deviation can be attributed to operator-specific measurement habits and operational workflows. It should be noted that these deviations represent the total geometric difference between the physical workpiece and nominal CAD geometry, incorporating both workpiece manufacturing deviations and measurement deviations. Comparisons across repeated tests show that the proposed automated inspection platform achieves consistent repeatability and reduces inspection time when the same surface coverage is maintained. It is important to clarify that CAD deviation maps capture geometric differences inherent to the workpiece, not the absolute accuracy of the measurement system. We plan to conduct additional validation using traceable standard artifacts in future work.

It should be emphasized that identical surface-coverage targets were predefined for both manual and automated measurement tests. The CAD deviation results in Figure 25 offer preliminary comparative insights into the measurement performance of the two inspection strategies. Nevertheless, these results alone are not adequate for rigorous validation of full measurement-quality equivalence. Comprehensive equivalence verification, including quantitative assessment of measurement bias and repeatability under identical coverage conditions, will be further explored in our follow-up research.

## 5. Conclusions

In response to the technical demands for automated inspection in the field of smart manufacturing, this paper designs a vision-based automated inspection platform. It conducts systematic research and experimental validation of its key technologies. First, the overall design of the automated inspection platform was completed. A rotatable fixture base was constructed to ensure stable and precise clamping and positioning of the parts under test. A robotic arm equipped with measurement devices was employed to overcome the limitations of fixed measurement angles. By combining a laser scanner and an optical tracker, multi-angle 3D data was acquired, and coordinate registration analysis between the measurement data and the standard digital model was established based on rigid-body matching theory. Specifications for the redundant placement of reference points were established to ensure the reliability of station transfer and coordinate registration. A two-level robotic arm trajectory planning method combining global coverage with local refinement was proposed, and collision simulation analysis was conducted to ensure interference-free motion throughout the workflow. Finally, a complete automated measurement system was built and experimentally validated. The experimental results demonstrate that the 3D vision-based automated inspection platform designed in this paper can efficiently perform automated measurement and analysis of workpieces. It exhibits favorable point-coordinate repeatability under fixed-clamping conditions, good versatility and adaptability for workpiece quality-monitoring tasks. Metrological dimensional accuracy validation against traceable standards, bias evaluation and full-scope uncertainty budgeting remain subjects for future research, and the platform can provide reliable technical support for online 3D automated inspection in smart-manufacturing scenarios.

## Figures and Tables

**Table 1:** Dobot CR5A robot specifications.

Brand	Model	Axes	Payload	Reach	Repeatability	Weight
Dobot	CR5A	6	5.0 kg	900 mm	0.02 mm	25 kg

**Table 2:** Robotic arm trajectory simulation process.

Start 1. Set initial target measurement points for global scanning (Global scan spacing: 2 mm). 2. Generate trajectories to fully cover the measured workpiece accordingly. 3. The robot performs simulation following the predefined target measurement points. 4. Collision detection analysis. (a) Global Trajectory Planning If collision or joint arm self-lock is detected; Move to the next target measurement point; If collisions still occur during detection using the initially configured target measurement points; The target measurement points will be re-planned; Collision simulation is then performed to carry out trajectory planning based on the newly defined target measurement points; Repeat until no collision and no self-lock occur; (Global planning termination rule: collision-free and no joint self-lock) (b) Local Trajectory Planning Detect high-curvature features from global scan data to trigger local refinement; (Refinement trigger: local surface curvature > 0.02 mm^−1^) Identify the local features to be measured; Set the starting target measurement point; Set the ending target measurement point; Determine scan spacing and scan count based on surface curvature; (Scan spacing decreases linearly with curvature, lower bound 0.10 mm; maximum scan lines for one local region: 20) Terminate local refinement upon full feature coverage or maximum scan count. (Termination condition: complete feature coverage or reaching the 20-line upper limit) 5. Confirm the final path. 6. Output the final trajectory code. End

**Table 3:** Initial measurement coordinates for fixed points (unit: mm).

Point ID	X	Y	Z	Point ID	X	Y	Z
1	−123.9442	25.1075	587.0378	6	19.4663	62.7615	553.2594
2	−103.9002	−68.4091	642.5198	7	24.4369	154.0404	494.8325
3	−89.8128	−124.3052	675.5088	8	119.2035	−9.5349	586.4405
4	−46.8741	145.8212	527.1480	9	138.8188	−118.8649	645.5516
5	14.1665	−63.1848	632.1124	10	133.2948	107.7195	508.9875

**Table 4:** Statistical results of repeated measurements for fixed target points (unit: mm).

Point ID	*u_x_*	*u_y_*	*u_z_*	*u_d_*	Point ID	*u_x_*	*u_y_*	*u_z_*	*u_d_*
1	0.0013	0.0035	0.0029	0.0023	6	0.0008	0.0037	0.0012	0.0019
2	0.0010	0.0039	0.0028	0.0024	7	0.0009	0.0032	0.0011	0.0015
3	0.0008	0.0040	0.0013	0.0020	8	0.0005	0.0034	0.0007	0.0016
4	0.0010	0.0034	0.0009	0.0016	9	0.0006	0.0036	0.0011	0.0018
5	0.0006	0.0035	0.0018	0.0016	10	0.0008	0.0036	0.0014	0.0019

**Table 5:** Comparison of inspection cycles for wheel-hub measurement.

Metrics	Manual Measurement	Automated Testing Platform
Scanning time/min	10	3.5
Fixture-motion time/min	0	1.5
Point cloud processing (filtering, registration)/min	10	3
Report generation/min	5	3
Total inspection cycle/min	25	11
Systematic error	Unstable manual scanning posture and personal measurement habits readily introduce errors.	Pre-planned scanning trajectory and fixture-fixed workpiece reduce human intervention.
Batch inspection	Time-consuming and high labor intensity	Template-based strategy enables batch inspection of identical workpieces

## Data Availability

The data presented in this study is available on request from the corresponding author. The data is not publicly available due to the ongoing confidential status of the research project.
